# Interactions of the emerging fungus *Candida auris* with *Acanthamoeba castellanii* reveal phenotypic changes with direct implications on the response to stress and virulence

**DOI:** 10.1128/spectrum.01746-24

**Published:** 2024-12-17

**Authors:** Michele Ramos Valente, Lucas Martins Alcântara, Deborah Santos Cintra, Susana Ruiz Mendoza, Elisa Gonçalves Medeiros, Kamilla Xavier Gomes, Leandro Honorato, Marcos de Abreu Almeida, Carmen Baur Vieira, Joshua Daniel Nosanchuk, Diana Bridon da Graça Sgarbi, Marcia Ribeiro Pinto, Leonardo Nimrichter, Allan Jefferson Guimarães

**Affiliations:** 1 Laboratório de Bioquímica e Imunologia das Micoses, Departamento de Microbiologia e Parasitologia, Universidade Federal Fluminense, Niterói, Rio de Janeiro, Brazil; 2 Programa de Pós-Graduação em Microbiologia e Parasitologia Aplicadas, Instituto Biomédico, Universidade Federal Fluminense, Niterói, Rio de Janeiro, Brazil; 3 Programa de Pós-Graduação em Imunologia e Inflamação, Universidade Federal do Rio de Janeiro (UFRJ), Rio de Janeiro, Brazil; 4 Laboratório de Glicobiologia de Eucariotos, Departamento de Microbiologia Geral, Instituto de Microbiologia Paulo de Góes, Universidade Federal do Rio de Janeiro (UFRJ), Rio de Janeiro, Brazil; 5 Núcleo de Pesquisa de Virologia, Departamento de Microbiologia e Parasitologia, Instituto Biomédico, Universidade Federal Fluminense, Niterói, Rio de Janeiro, Brazil; 6 Infectious Diseases, Albert Einstein College of Medicine, Yeshiva University, Bronx, New York, USA; 7 Rede Micologia RJ – Fundação de Amparo à Pesquisa do Estado do Rio de Janeiro (FAPERJ), Rio de Janeiro, Brazil; 8 National Institute of Science and Technology (INCT) in Human Pathogenic Fungi, São Paulo, Brazil; Barnard College, New York, New York, USA

**Keywords:** *Acanthamoeba castellanii*, *Candida auris*, interactions, virulence, resistance

## Abstract

*Candida auris* is an emerging fungal pathogen notable for its resistance to multiple antifungals and ability to survive in various environments. Understanding the interactions between *C. auris* and environmental protozoa, such as *Acanthamoeba castellanii,* could provide insights into fungal adaptability and pathogenicity. Two *C. auris* isolates (MMC1 and MMC2) were co-cultured with *A. castellanii* to examine interaction dynamics, survival, stress responses, growth, virulence, biofilm formation, and antifungal susceptibility. The association *of C. auris-A. castellanii* varied with a multiplicity of infection (MOI), with MMC2 exhibiting higher association rates at increased MOI than MMC1. Both isolates survived distinctly within *A. castellanii*, as the MMC1 showed an initial decline and subsequent increase in viability, while MMC2 maintained higher viability for up to 24 h, decreasing afterward. Both isolates exhibited accelerated growth when recovered from *A. castellanii*. The MMC2 isolate displayed increased resistance to oxidative, osmotic, and thermal stresses upon interaction with *A. castellanii*, whereas MMC1 showed limited changes. Exposure to *A. castellanii* also influenced the expression of virulence factors differently, with MMC1 increasing phospholipase and peptidase, while MMC2 upregulated phytase, esterase, hemolysin, and siderophores. Upon contact with *A. castellanii*, MMC2 enhanced biofilm formation, unlike MMC1. Both isolates increased ergosterol upon interactions, enhancing susceptibility to amphotericin B. However, both isolates were more tolerant to itraconazole and caspofungin, particularly MMC2, which showed differential expression of ergosterol biosynthesis enzymes and increased cell wall polysaccharides. This study reveals that interactions with *A. castellanii* modulate *C. auris* physiology and virulence, contributing to its environmental adaptability and resistance to antifungals.

**IMPORTANCE:**

*Candida auris* has emerged as a critical public health concern due to its resistance to multiple antifungal drugs and ability to survive on surfaces under harsh conditions, mainly due to biofilm formation. The precise origin of this emerging pathogen still awaits elucidation, but interactions with environmental protozoa may have helped *C. auris* to develop such virulence and resistance traits. In this work, we precisely characterize the interactions of *C. auris* with the free-living amoeba *Acanthamoeba castellanii* and how these protozoa may alter the fungal behavior in terms of virulence, thermotolerance, biofilm formation capacity, and drug resistance. It may be essential to understand the various interactions *C. auris* could perform in the environment, directly impacting the outcome of human infections under the One Health approach.

## INTRODUCTION

There are an estimated 1.5 million deaths each year attributed to fungal disease (1). Although *Candida albicans* continues to be a major cause of morbidity and mortality, the past 2 decades have seen an upsurge in the incidence of non-*C*. *albicans* species (2). Notably, *Candida auris*, initially identified in Japan in 2009 along with early documented cases in Asia, represents an emerging multidrug-resistant fungal pathogen that has garnered significant attention due to its global spread and remarkable clinical impact (3). Since its discovery, *C. auris* has been extensively and rapidly disseminated across different continents, including Europe, the Americas, Africa, and Australia. This has led to the cessation of updates to the Centers for Disease Control and Prevention epidemiology map (4, 5).

Among the various species capable of causing infections in humans, *C. auris* stands out due to its distinctive characteristics, including its propensity for multidrug resistance to clinically used antifungals, its extreme resistance to disinfectants, and its ability to persist on surfaces, all of which facilitate its ability to cause severe infections (6–8). Its survival ability in healthcare settings, including hospitals, nursing homes, and intensive care units, has significantly contributed to its widespread transmission and emergence as a prominent healthcare-associated pathogen. According to WHO, it is now ranked among the top 5 fungal pathogens posing the most urgent threat to public health (9–11).

*C. auris* harbors numerous virulence factors that contribute to its pathogenicity. These factors encompass the production/secretion of extracellular enzymes, including proteases and lipases, which facilitate tissue invasion and immune evasion (7, 12). Furthermore, *C. auris* forms drug-resistant biofilms, enhancing its resistance to antifungals and complicating treatment (2, 6). The elevated mortality rates associated with *C. auris* infections, coupled with limited treatment options, underscore its clinical significance and the urgent need for effective management strategies (13, 14).

The precise origin of *C. auris* remains uncertain (15). It is hypothesized that the emergence of this species initially occurred in environmental contexts prior to clinical recognition, adapting to become pathogenic to humans under stressful conditions (16). Indeed, *C. auris* exhibits remarkable environmental adaptability, allowing its persistence beyond clinical settings (15). Among the stressful conditions, its thermal resilience in response to climate changes is noteworthy (17). Factors supporting this theory include the identification of *C. auris* isolates at two environmental sites in the Andaman Islands (18). Clinically, this species is predominantly encountered in cooler skin locations rather than the gastrointestinal tract, although its optimal growth is at 42°C (17). Furthermore, it has been detected in various environmental niches, encompassing water sources, soil, and even vegetation (8, 17).

These environmental reservoirs potentially act as vectors for the dissemination of *C. auris* and might contribute to its geographical spread and ongoing transmission, thereby complicating the efforts to eradicate the fungus from healthcare facilities (8). Due to the potential of the environment to serve as a boot camp for the development and enhancement of *C. auris* virulence, the interaction of *C. auris* with environmental hosts, including insects and other organisms, remains an active area of investigation (19). Nevertheless, it is acknowledged that survival, both within hosts and in natural environments, involves exposure to conditions characterized by significant temperature fluctuations, extreme humidity, and predation by other microorganisms, which could select traits that enhance virulence (8, 16, 17, 19).

In that sense, the free-living amoeba (FLA) *Acanthamoeba castellanii,* besides being an opportunistic pathogen known to cause corneal, central nervous system, and skin infections (20, 21), due to its ubiquitous distribution, could also serve as an environmental host and reservoir for important pathogens, favoring increased intracellular resistance, and thus representing an excellent model for study (20, 22). Research has reported the survival of pathogenic fungi such as *Histoplasma capsulatum* (23, 24), *Paracoccidioides* spp. (24, 25), *Cryptococcus* spp. (24, 26, 27), and *Candida* spp. (24, 28, 29) within *A. castellanii,* resulting in increased fungal virulence, prompting our investigation into such mechanisms with *C. auris*.

Given that *A. castellanii* is one of the mainly studied mycophagous FLAs and considering the potential for environmental encounters between *C. auris* and *A. castellanii*, this study aimed to elucidate the interactions between these emerging pathogens. Our focus included analyzing their association dynamics and phenotypic plasticity of *C. auris*, including its survival, growth, stress response, antifungal susceptibility, and virulence modulation through its interaction with *A. castellanii*. Beyond the direct physical contact, cellular by-products of *A. castellanii*, such as released components (from conditioned supernatant) and its extracellular vesicles (EVs), may serve as mediators of intercellular communication fungal-amoeba, potentially influencing *C. auris* physiology (30–32). As *C. auris* exemplifies the One Health concept, which underscores the interconnectedness of human, animal, and environmental health (33), comprehending the dynamics of *C. auris* in the environment and its interactions with environmental hosts is crucial for developing effective control strategies for this emerging pathogen.

## MATERIALS AND METHODS

### Cultivation of *Candida auris*

*C. auris* MMC1 and MMC2 are fluconazole-resistant and sensitive clinical isolates, respectively (34). *C. auris* yeast cells were cultured on Sabouraud plates at 37°C for 48 h. Subsequently, a colony was randomly selected, inoculated into 20 mL Sabouraud liquid medium, and cultured for 24 h at 37°C with agitation at 150 rpm. Yeasts were then centrifuged at 1,100 *g* for 10 min, washed three times with an excess of phosphate-buffered saline (PBS; 140 mM NaCl, 2.7 mM KCl, 1.5 mM KH_2_PO_4_, 17 mM Na_2_HPO_4_, pH 7.2), suspended in PBS, and enumerated using a hemocytometer.

### Cultivation of *A. castellanii*

The environmental reference strain ATCC 30010 (Neff) of *A. castellanii* was acquired from the American Type Culture Collection (VA, USA). Trophozoites were cultured in peptone-yeast extract-glucose (PYG) medium containing 100 mM glucose, 0.4 mM CaCl_2_, 0.4 mM MgSO_4_, 2.5 mM Na_2_HPO_4_, 2.5 mM KH_2_PO_4_, 1 g/L sodium citrate, and 0.05 mM Fe(NH4)_2_(SO_4_)_2_, adjusted to pH 6.5 (24). *A. castellanii* trophozoites were grown in 175 cm^2^ culture flasks at 28°C for approximately 48 h until reaching confluence, achieving viability of approximately 100%, as checked by Trypan blue staining (35).

### Extraction of *A. castellanii* secretome

*A. castellanii* trophozoites were decanted from the culture supernatant by centrifugation at 2,000 × *g* for 10 min. The supernatants were then centrifuged at 15,000 × *g* for 10 min to ensure the removal of any remaining cellular debris. The resulting sediments were discarded, and supernatants were concentrated 20-fold by ultrafiltration using an Amicon (cutoff = 10 kDa, Merck). The concentrated supernatants were subsequently centrifuged at 100,000 × *g* for 1 h at 4°C, and the resulting supernatant was collected as EV-free supernatant. The remaining sediments were washed twice with PBS through sequential centrifugations and suspensions to obtain the particulate secretome containing *A. castellanii* EVs (31, 32). Protein concentration in both samples was determined using the bicinchoninic acid method following the manufacturer’s instructions (Thermo Scientific), and samples were stored at −80°C until used.

### Analysis of time and multiplicity of infection in the interaction kinetics of *A. castellanii* with *C. auris*

Cultured trophozoites were mechanically detached from the flasks, centrifuged at 800 *g* for 10 min, suspended in fresh PYG, and counted. Then, 800 µL of a 10^6^ trophozoites/mL suspension were plated into each well of 24-well plates. Plates were then incubated for 1 h at 28°C, as the optimal temperature for the growth of *A. castellanii* trophozoites (24, 30–32, 36, 37), and does not compromise the viability of *C. auris. C. auris* yeasts (MMC1 and MMC2 *C. auris* isolates), previously washed with PBS, were incubated with 40 µg/mL NHS-Rhodamine (Thermo Scientific) for 30 min at room temperature (RT) (38). After five washes with excess PBS, yeasts were suspended in fresh PYG, enumerated using a hemocytometer, and added to *A. castellanii* at different multiplicity of infections (MOI; 10 yeasts: 1 *Acanthamoeba*, 5:1, 2:1, 1:1). The plates were incubated at 28°C for 1, 2, or 3 h. Wells were washed to remove non-interacting *C. auris* yeasts, and trophozoites were detached and analyzed by flow cytometry (FACSCalibur, BD) for quantification and determination of the percentage of trophozoites containing associated fungi (adhered or internalized), designated as positive for the FL2-channel (%FL2^+^). The experiments were repeated three times with two biological replicates. The average percentage of interactions for the two variable combinations was calculated, and values were recorded. We then established the mathematical parameters of simple derivation to characterize the true influence of each variable. Through the more precise dynamic fitting of the points, the following plane equation was used for comparison between time and ln(MOI) inclinations in the SigmaPlot software version 13.0 (Systat Software, Inc., TN, USA), with *P* < 0.05 values considered statistically significant for the influence of each variable on the interaction rate:


Interaction rate (%)=a x time+b xln⁡(MOI)+c

*P* < 0.05 values were considered statistically significant for the influence of each variable on the fungus-amoeba interaction rate.

### Characterization of *C. auris* viability and generation time after interaction with *A. castellanii*

*A. castellanii* trophozoites were obtained as described above, and 200 µL of 10^6^ trophozoites/mL suspension were plated per well of a 96-well flat-bottom microplate. After 1 h of incubation for trophozoite adhesion, *C. auris* yeasts (MMC1 and MMC2 isolates) were suspended in PYG medium and added at an MOI of two yeasts:one amoeba. Plates were then incubated for different intervals (3 h, 12 h, 24 h, and 48 h) at 28°C, washed to remove non-interacting yeasts, and amoebae were lysed with cold sterile water for 30 min at RT. Recovered yeasts were counted, diluted in PBS, and plated on Sabouraud agar medium (37). As a control of yeast viability, *C. auris* was incubated in PYG medium without *A. castellanii*. After incubations for 48 h at 37°C for *C. auris* growth, colony-forming units (CFUs) were counted and compared between treatments. The percentage viability at each time point was calculated as the total CFUs divided by the counted number of yeasts for controls and *C. auris* in contact with *A. castellanii*.

### Direct and indirect interactions of *A. castellanii* with *C. auris*

*A. castellanii* trophozoites were obtained as described above, and 800 µL of a 10^6^ trophozoites/mL suspension were plated per well of a 24-well plate. After 1 h, supernatants were removed from each well, and MMC1 and MMC2 *C. auris* isolates (previously washed and suspended in PYG medium) were added at one yeast:one amoeba. Plates were incubated at 28°C for the established 48-h co-incubation period. For conditions of indirect interaction, *C. auris* were washed, and an equal number of *C. auris* was incubated with PYG in the presence of 20 µg/mL of *A. castellanii* EVs in PYG. As an additional condition of indirect interaction, *C. auris* yeasts were washed and suspended in conditioned *A. castellanii* supernatant after 48 h of growth instead of PYG, in the same quantity described above. As a control, *C. auris* was incubated for 48 h only with PYG medium.

### Assessment of *C. auris* growth rate after interaction with *A. castellanii*

For the determination of the viability and growth capacity of both MMC1 and MMC2 isolates of *C. auris* upon interactions with *A. castellanii*, under all aforementioned conditions after 48 h of incubation, the well contents were transferred to 15 mL conical tubes, kept on ice, and centrifuged at 1,100 × *g* for 10 min. Subsequently, the supernatant was discarded, and sediments were suspended in distilled water and kept at 4°C for 30 min for amoeba lysis. Then, *C. auris* yeasts from both isolates and treatments consisting of direct interactions with *A. castellanii* or indirectly with its by-products, as well as cells that did not undergo interaction (PYG control), were washed three times with PBS and enumerated using a hemocytometer. Yeasts were diluted to 2.5 × 10^3^ cells/mL in Roswell Park Memorial Institute 1640 (RPMI) medium. Subsequently, 200 µL of the yeast suspension was added to the wells of a 96-well microplate, which were incubated at 37°C. Plate absorbances were read at 600 nm every 6 h for 48 h, and values were used to construct and compare the *C. auris* growth curves under each condition as described (39, 40). Generation (doubling) times in the log phase were calculated using the following equation:


$$Doubling\;time=\frac{\left(t_{n}-t_{0})*ln(2\right)}{ln(Abs_{tn}/Abs_{t0})}$$

### Sensitivity of *C. auris* to various stress conditions after interaction with *A. castellanii*

The susceptibility of *C. auris* cells recovered upon the described treatments (direct interactions with *A. castellanii*, indirect interactions with *A. castellanii* secreted fractions or PYG-only control) was tested on Sabouraud plates containing stress-inducing factors: NaCl (1.5 M), sorbitol (1 M), H_2_O_2_ (2.5 mM), pH levels (2.0, 4.0, 7.0, and 10.0), and temperatures (28°C, 37°C, and 42°C). Initially, *C. auris* suspensions corresponding to the three conditions were diluted with PBS to an optical density at 600 nm of 0.1, transferred to 96-well plates, and then serially diluted at 1:10. Aliquots (2.5 µL) of the diluted suspensions were plated (10^−1^ to 10^−5^). Plates were incubated at either 28°C, 37°C, or 42°C for 3 days. For additional evaluations of resistance to oxidative stress, the recovered *C. auris* was plated in Sabouraud plates with H_2_O_2_ (2.5 mM) and incubated for 48 h at 37°C, and CFUs were counted and compared among groups. Experiments were repeated three times.

### Evaluation of biofilm formation by *C. auris* after interactions with *A. castellanii*

Following the conditions described (direct interactions with *A. castellanii*, indirect interactions with *A. castellanii* secreted fractions, including conditioned supernatant and EVs, or PYG-only control), *C. auris* was suspended at 1 × 10^6^ yeasts/mL in RPMI, consistent with the optimal concentration previously reported for biofilm formation (41, 42). Aliquots of 300 µL were added for biofilm growth. Culture medium-only was used as a negative control. After cultivation at intervals of 24 and 48 h, biofilm formation was assessed using crystal violet for spectrophotometric evaluation of biomass (492 nm) and Alamar blue (formazan) for analysis of the biofilm metabolic activity (570 nm).

### Detection of extracellular enzymes and metabolic activities of *C. auris* after interaction *with A. castellanii*

The detection of hydrolytic enzymes and siderophore activities were performed upon the distinct treatments (direct interactions with *A. castellanii*, indirect interactions with *A. castellanii* secreted fractions, including conditioned supernatant and EVs, or PYG-only control). Suspensions of 5 µL of 2 × 10^8^ yeast cells/mL were added to Petri dishes containing media/substrates specific to different enzymes at 37°C for 7 days. The enzymatic activity (peptidase, esterase, phytase, phospholipase, hemolysin) and siderophore levels were assessed by measuring the clear (or precipitation) halo around the fungal colony and dividing by the diameter of the colony. The plates were prepared using the standard mediums described (Table 1).

**TABLE 1:** Standard mediums used for the evaluation of *C. auris* secreted enzymes and siderophores

Activity	Measurement	Reference
Phospholipase	Precipitation (dense white) halo	Egg yolk assay (43)
Phytase	Degradation (translucent) halo	Calcium phytate (44)
Esterase	Precipitation (dense white) halo	Tween 80 assay (45)
Peptidases	Degradation (translucent) halo	Bovine serum albumin (BSA) hydrolysis. Adapted from reference (46)
Hemolysin	Degradation (translucent) halo	Hemolytic activity (47)
Siderophores	Degradation (yellowish) halo	Chrome Azurol S (CAS) agar plates (48)

### Antifungals sensitivity testing of *C. auris* after interaction with *A. castellanii*

The evaluation of *C. auris* sensitivity to antifungals followed the standard methodology recommended by the Clinical and Laboratory Standards Institute (protocol M27-A2) (49). After isolation of MMC1 and MMC2 *C. auris* from the distinct treatments (direct interactions with *A. castellanii*, indirect interactions with *A. castellanii* secreted fractions, including conditioned supernatant and EVs, or PYG-only control), yeasts were washed in PBS and diluted to a concentration of 5 × 10^3^ cells/mL in RPMI medium, pH 7.0, buffered with 3-(N-morpholino)propanesulfonic acid (MOPS) at 0.165 mol/L. Simultaneously, 1:2 serial dilutions of different antifungals (amphotericin B [polyene], itraconazole [azole], and caspofungin [echinocandin]) were made in a volume of 100 µL of RPMI-MOPS in 96-well plates, in a range of 32 μg/mL–0.032 μg/mL. Controls included wells without antifungals. Subsequently, 100 µL of the yeast suspension was added to each well, and plates were incubated at 37°C with shaking at 150 rpm. Plate absorbances were read at 600 nm every 3 h for 72 h. The evaluations were performed twice with two independent biological replicates each.

### Assessment of *C. auris* gene expression after contact with *A. castellanii*

The MMC1 and MMC2 *C. auris* isolates were recovered upon interaction with trophozoites of *A. castellanii* or PYG controls*,* as described above. Yeasts were washed in cold PBS, and RNA was isolated using the TōTALLY RNA Kit (Life Technologies). Extracted RNA was further treated with DNAse, and the concentrations were determined in a NanoDrop spectrophotometer (NanoDrop Technologies). RNA integrity was evaluated by electrophoresis, and sample purity was confirmed by a 260/280 absorbance ratio of >1.8. The cDNA synthesis was performed using 1 µg of RNA with the ImProm-II Reverse Transcription System (Promega, WI, USA). The quantitative reverse transcription polymerase chain reactions (RT-qPCR) were carried out in the 7500 Fast Real-Time PCR System (Applied Biosystems) using the PowerTrack SYBR Green Master Mix for qPCR (ThermoFisher), for evaluating the expression of genes Erg 6, Erg 11, Fks1p, Fks2p, Hog1, and tubulin (Table 2). These genes were selected based on their involvement in several resistance mechanisms to the antifungal drugs tested. Erg 11 is the target for azole drugs, and its overexpression leads to increased azole tolerance (50). Erg 6 is also involved in the alternative ergosterol synthesis pathway that avoids the accumulation of toxic sterol intermediates upon azole ergosterol biosynthesis inhibition (50). Hog1 is involved in the response to stressors, and its absence is related to increased resistance to harsh conditions and caspofungin. The Fks1p and Fks2p are enzymes involved in the β-1,3-glucan production, fungal cell wall assembly, and biofilm matrix development, and their overexpression has been described in some *Candida* species (51–53). In each well of a MicroAmp Fast 96-well reaction plate, the qPCR mix consisted of 5 µL PowerTrack SYBR Green Master Mix, 0.5 µL Yellow Sample buffer, 0.8 µM of forward and reverse primers (Table 2), 50 ng cDNA, and nuclease-free water to a final volume of 10 µL. The *C. auris* tubulin was elected as housekeeping for normalizations of expressions as described (54). Negative controls were performed with no addition of cDNA templates. The qPCR thermal cycling conditions consisted of an initial denaturation step at 95°C for 10 min, then 40 cycles of 95°C for 15 s, and 60°C for 1 min. At the end of the RT-qPCR, melting curves were performed for individual samples by increasing the temperature from 65 to 95°C at a rate of 0.1°C/s. Experiments were performed in triplicate, with two experimental replicates each.

**TABLE 2:** Real-time PCR primers used for amplification of the Erg 6 (OK564601.1), Erg 11 (MK294634.1), Fks1p (MK059974.1), Fks2p (XM_054702049.1), and Hog 1 (XM_029036387.2)^*a*^

Target	Primers	Sequences (5´−3´)	Length	Start	Stop	Tm (°C)	GC%	Product length
Erg 6	Forward	CGTTGGGCGACTACTTCACT	20	908	927	60.04	55	116
	Reverse	GACCTGCTTGGACCCCTTTG	20	1,004	1,023	60.90	60	
Erg 11	Forward	CGCTAAGCTTGCGGATGTTT	20	318	337	59.60	50	57
	Reverse	ACTGGAGTGGTCAAGTGGGAAT	22	353	374	61.30	50	
Fks1p	Forward	TGGTAGATTCATCGCCGACA	20	5,478	5,497	58.89	50	115
	Reverse	GACCACCAGGATGAGACCTT	20	5,572	5,591	58.72	55	
Fks2p	Forward	TACACCCAGCAACGCAATTT	20	5,574	5,593	59.54	45	153
	Reverse	GCTGCGATTAAGCTGGGAAA	20	5,707	5,726	58.91	50	
Hog1	Forward	CAGAGACCATGTGCACCAGT	20	657	676	59.96	55	298
	Reverse	CTCGCAAATTGGCTCATCGG	20	935	954	59.97	55	
Tubulin	Forward	GAGAGAGGCCGAAGGTTGTG	20	363	382	60.40	50	60
	Reverse	CCACCCAAGGAGTGAGTAATCTG	23	400	422	62.10	52.2	

^*a*^ The tubulin (XM_029035310.1) was elected as the housekeeping for transcript normalization (51, 54).

### Extraction of *C. auris* sterols after contact with *A. castellanii*

Both *C. auris* isolates recovered from the distinct incubations described above (direct interactions with *A. castellanii*, indirect interactions with *A. castellanii* secreted fractions, including conditioned supernatant and EVs, or PYG-only control) were used to extract fungal sterols, following the Folch method with slight modifications (55). Additionally, to verify the longevity of sterol increase in *C. auris*, yeasts recovered from amoeba were subcultured every 48 h for six passages in PYG medium, and the cells were reserved. *C. auris* was recovered after washing with PBS at 4°C and enumerated, and approximately 10^8^ cells were added to 1.5 mL of chloroform:methanol (C:M) in a 2:1 (vol/vol) ratio. The suspensions were then vortexed for 30 s and incubated for 1 h at RT. Subsequently, yeasts were centrifuged at 1,100 × *g* for 10 min, and the supernatants were recovered. Two additional extraction steps were carried out with chloroform:methanol in a 1:1 (vol/vol) and 1:2 (vol/vol) ratio, and the resulting supernatants were combined. After extraction, the contents were evaporated with N_2_, and the dried material was washed with 2 mL of chloroform, 1 mL of methanol, and 0.5 mL of 0.47% KCl. The resulting volumes were collected, combined, and vortexed for 30 s. After incubation for 1 h at RT, the samples were centrifuged at 1,100 × *g*, and the lower phase (LP) was recovered from each sample. After drying with N_2_, the LP was suspended in C:M (1:1). The samples (5 µL) were applied to a thin-layer chromatography (TLC) silica gel 60 plates (Merck). The TLC was performed using cyclohexane:ethyl acetate in a 3:2 (vol/vol) ratio as the mobile phase, and plates were revealed by spraying with a solution composed of 50 mg of FeCl_3_, 5% (vol/vol) of acetic acid, and 5% (vol/vol) of sulfuric acid followed by heating to 25°C for 5 min–10 min (56).

### Assessment of *C*. *auris* cell wall polysaccharides after incubations with *A. castellanii*

Both isolates (MMC1 and MMC2) of *C. auris* recovered from the interactions with *A. castellanii* or controls (direct interactions with *A. castellanii*, indirect interactions with *A. castellanii* secreted fractions, including conditioned supernatant and EVs, or PYG-only control) were washed (1,100 × g/10 min) and fixed with 4% paraformaldehyde in PBS for 20 min at RT. Then, yeasts were washed and incubated with 5 µg/mL of Dectin-1-Fc(IgG2b) diluted in 1% bovine serum albumin (BSA) prepared in PBS for 1 h at RT. After washes, yeasts were incubated with 1 µg/mL of an anti-mouse Alexa Fluor 488 conjugate (Thermo Scientific) and washed thrice in PBS. FL1 fluorescence intensity of yeasts was evaluated by flow cytometry (FACSCalibur, BD). Alternatively, chitin levels were assessed by staining the cells with Uvitex2B, and mannosylated component levels were evaluated using Concanavalin A-TRITC as described (24, 36, 37).

### Assessment of *C. auris* virulence after incubation with *A. castellanii*

*C. auris* recovered from the interactions with *A. castellanii* or controls (direct interactions with *A. castellanii*, indirect interactions with *A. castellanii* secreted fractions, including conditioned supernatant and EVs, or PYG-only control) were washed with PBS and enumerated in a hemocytometer. Selected larvae of the lepidopteran *Galleria mellonella* (approximately 100 mg) were infected through the last left pro-leg with a 10 µL inoculum of 10^8^ yeasts/mL using a 0.701N 10 µL Hamilton syringe. Infected larvae (*n* = 10/group) were placed in Petri dishes and kept at 37°C (57, 58). Kaplan-Meier survival curves of the larvae were constructed for infections with fungi recovered from amoeba, treated and untreated with the *A. castellanii* secretome, and sham controls, and the groups were compared.

### Statistical analyses

Statistical analyses were performed using GraphPad Prism 9. Comparisons between groups were made by one-way analysis of variance. For post-tests, the Dunnett’s test was used for individual comparison of each group with the control, while Tukey’s was used for pairwise comparisons between groups. Each experiment was repeated at least three times. For survival analysis, curves were compared using the Log-rank (Mantel-Cox) test. For all analyses, *P* ≤ 0.05 was considered statistically significant.

## RESULTS

### *C. auris* isolates differentially interact with *A. castellanii*

**Fig 1:**
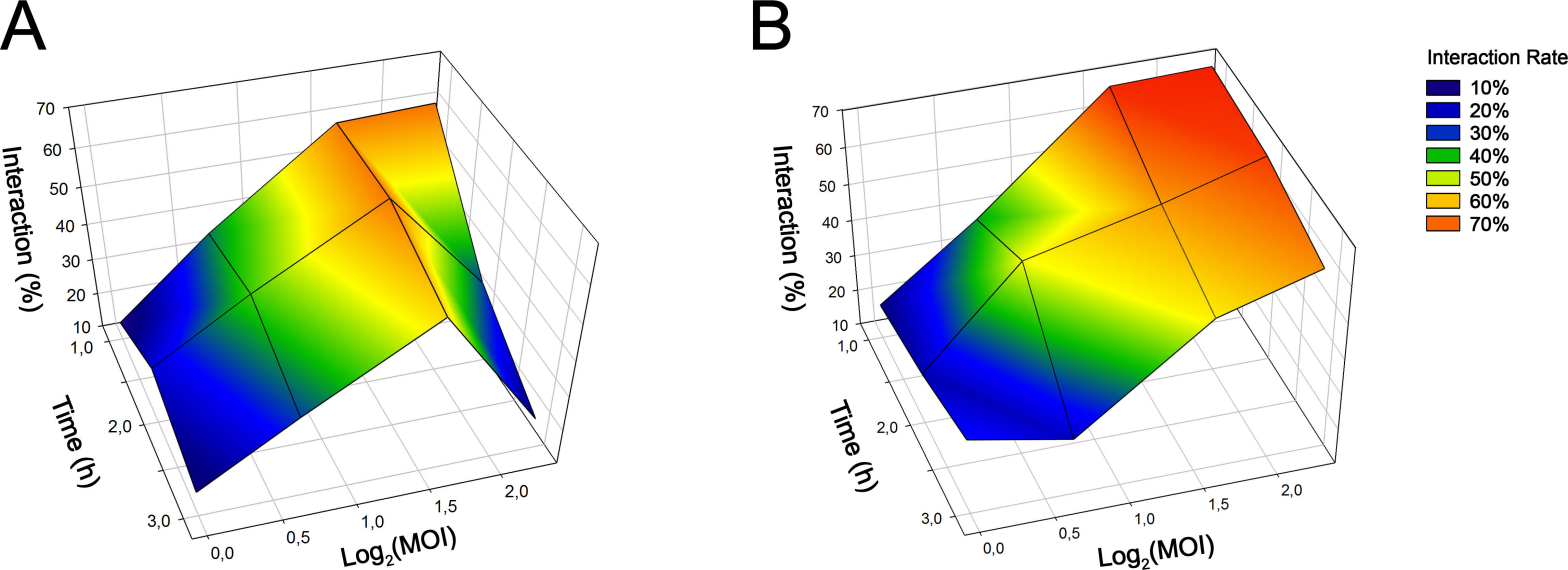
3D mesh heat plots illustrating the average percentage of interactions (z-axis) performed in triplicate, between *C. auris* MMC1 (left panel) or MMC2 (right panel) yeasts with *A. castellanii* at various time intervals (1 h, 2 h, and 3 h, y-axis) across different MOI (10:1, 5:1, 2:1, and 1:1, x-axis), with each node representing a single combination of the two variables. The natural logarithm of the MOI was applied to linearize the MOI scale. The impact of each variable on the interaction kinetics between *C. auris* and *A. castellanii* was assessed through simple derivation of the mathematical parameters and determination of the best trend plane via dynamic fit using SigmaPlot. Statistical significance of the variable slopes was achieved when *P* < 0.05.

Both *C. auris* isolates were co-cultured with *A. castellanii* to assess the effects of time and MOI variables on the magnitude of the fungus-amoeba association. Specifically, the percentages (%) of FL2^+^ trophozoites interacting with *C. auris* at various combinations of time/MOI were examined, and a 3D mesh heat plot was constructed for each *C. auris* isolate, MMC1 and MMC2 (Fig. 1A and B, respectively). A mathematical analysis was then performed by simple derivation of the best trend plane through a dynamic fitting of the data points (equation (1) and Table 3). For both isolates, the time variable did not statistically impact the fungus-amoeba interaction (Table 3). Regarding the ln(MOI) variable, although a proportional impact on the interaction was observed for the MMC1 isolate (9.94; *P* = 0.077), no statistical significance was reached (Fig. 1A). In contrast, for the MMC2 isolate, the ln(MOI) variable strongly impacted the *C. auris*-amoeba interaction (18.5; *P* < 0.0001; Fig. 1B).

**TABLE 3:** Best trend plane equation derived from dynamic fitting to compare the time and ln(MOI) slopes, aiming to determine the importance of each parameter in the interaction between *A. castellanii* and (A) MMC1 and (B) MMC2 *C. auris* isolates

*C. auris* × *Acanthamoeba castellanii*	Equation (*Z* = *a* × *X* + *b* × *Y* + *Z*) *X* = time *Y* = ln(MOI)	Slopes	
		A (time)	B (ln MOI)
MMC1	*Z* = −6.28 × time + 9.94 × ln(MOI) + 39.3	−6.28 (*P* = 0.27)	9.94 (*P* = 0.077)^*a*^
MMC2	*Z* = −3.33 × time + 18.5 × ln(MOI) + 33,0	−3,3 (*P* = 0.26)	18.5 (*P* < 0.0001)

^*a*^ Underlined slope values indicate statistical significance (P < 0.05).

### Viability of *C. auris* upon interaction with *A. castellanii*

**Fig 2:**
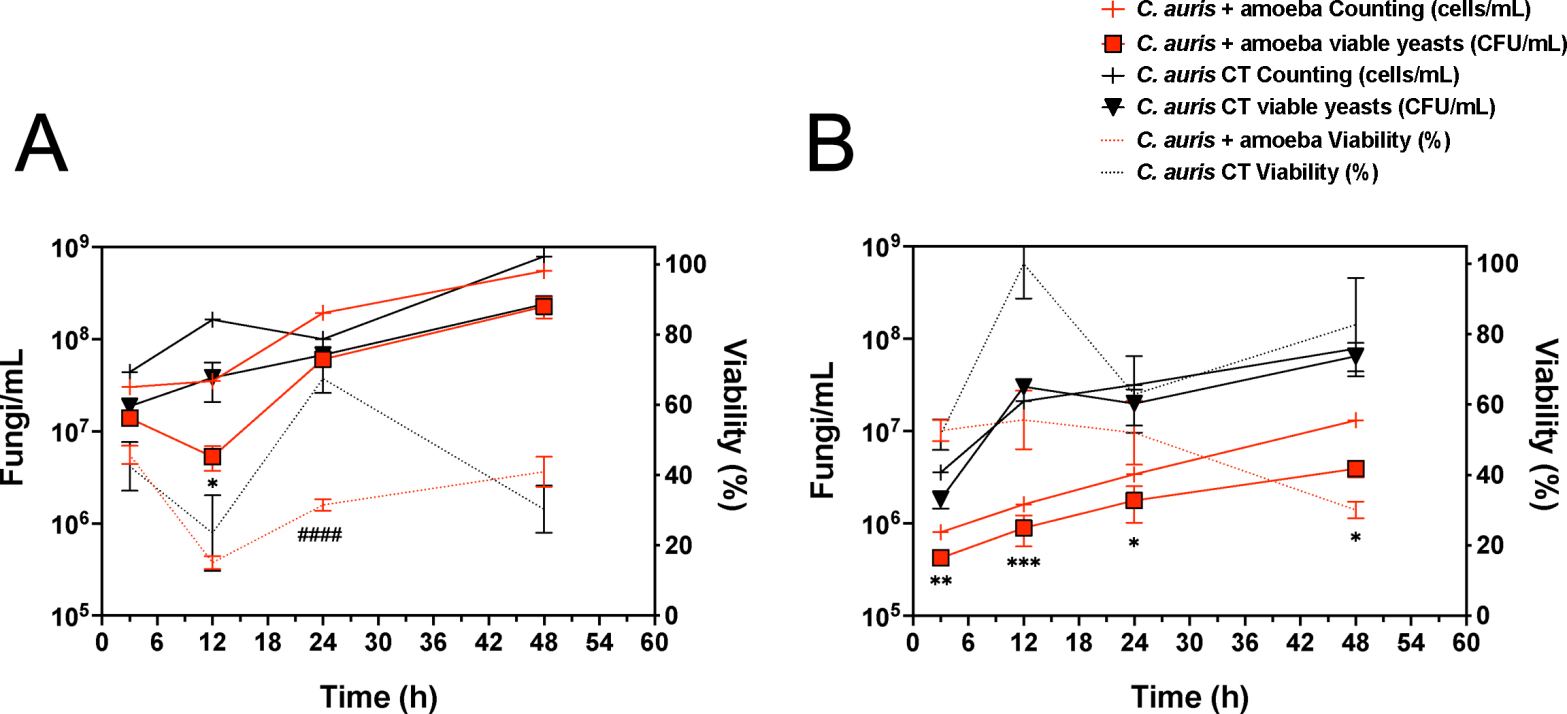
Assessment of *C. auris* yeast growth and viability following entry (2-h incubation) to *A. castellanii* at distinct time points (0 to 48 h) for the (**A**) MMC1 and (**B**) MMC2 isolates of *C. auris*. The number of fungi/mL is depicted on the left y-axis, representing yeasts counted in the hemocytometer in medium alone (control, black lines with crosses) and upon interaction with *A. castellanii* (red lines with crosses), compared to the number of viable yeasts (CFU/mL) in medium alone (black lines with inverted triangles) and upon interactions with *A. castellanii* (red lines with inverted triangles). Statistical analysis demonstrates the difference in CFU/mL averages between the PYG medium controls and the viable yeast recovered upon interaction with *A. castellanii* (*P < 0.05, **P < 0.01, and ***P < 0.001). The percentage of viability (right y-axis) over time, calculated by the ratio of CFU × 100/(counted cells), is represented as a black dashed line for PYG medium controls and a red dashed line for interactions with *A. castellanii* (####*P* < 0.0001). The experiments were performed in biological triplicate, with two experimental replicates each.

To assess the *C. auris* adaptability to the intracellular environment of *A. castellanii*, both MMC1 and MMC2 isolates of *C. auris* were recovered following interactions with amoeba. After co-culture, the yeast cells were plated, incubated, and then counted to determine the number of viable yeast cells after contact with the environmental predator. Both isolates were able to survive within the *A. castellanii* intracellular milieu and also exhibited distinct behaviors. The MMC1 isolate initially had a 46% viability, which decreased to 15% within 12 h, suggesting that *A. castellanii* could partially combat the infection. However, after 24 and 48 h, respectively, the percentage of viable fungi increased to 32% and 41%, indicating fungal growth (Fig. 2A). Conversely, the MMC2 isolate maintained a viability of over 50% up to 24 h, decreasing to 30% within 48 h of co-incubation (Fig. 2B). Therefore, we established a 48-h co-incubation period of *C. auris* and *A. castellanii* for the remaining experiments, where we could still detect an increase in CFUs for both MMC1 and MMC2 isolates of *C. auris*.

### Characterization of the growth curves of *C. auris* upon direct or indirect interactions with *A. castellanii*

For both MMC1 and MMC2 isolates, *C. auris* recovered after direct mechanical interaction with *A. castellanii* or exposure to its secreted products (conditioned supernatant) were cultivated in Dulbecco’s modified Eagle medium (DMEM), and their growth was compared to *C. auris* cultured in PYG medium (control). Overall, no differences in total growth were observed for either isolate for the different treatment conditions (Fig. S1A and B, respectively; *P* > 0.05). However, analysis of the slope of the curves comprising the exponential growth phase revealed contact with *A. castellanii* or its secreted products caused the MMC1 isolate to double at approximately 3.6 h and 3.7 h, respectively, compared to the 5.5 h for the control MMC1 *C. auris* in PYG medium. Similarly, upon contact with *A. castellanii* or its secreted products, the MMC2 isolate exhibited a doubling time of every 2.6 h and 2.5 h, respectively, compared to the 4.7 h for the control *C. auris* in PYG medium (Fig. S1C). Overall, the increased lag time for the groups of direct and indirect interaction fungus-amoeba is consistent with the modest increase in doubling time during the log phase.

### *C. auris* response to oxidative and osmotic stress upon direct or indirect interactions with *A. castellanii*

**Fig 3:**
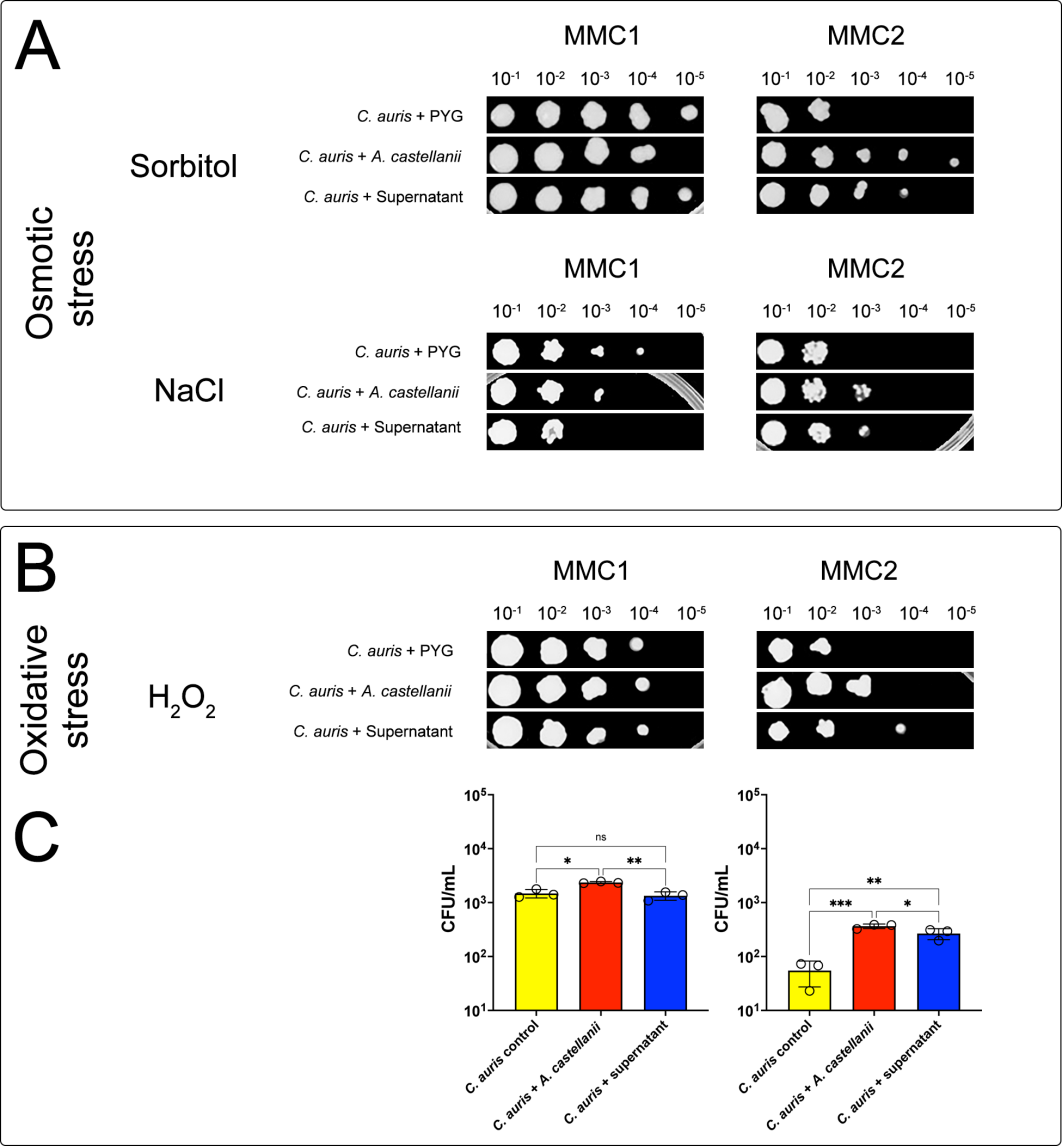
Spot assay for evaluating oxidative and osmotic stress sensitivity of *C. auris* following contact with *A. castellanii* trophozoites or its fractions. After the distinct treatments (direct mechanical interaction with *A. castellanii*, exposure to its secreted products-conditioned supernatant) or controls), recovered yeast cells from each condition were normalized to 0.1 absorbance at 600 nm, further serially diluted 10-folds (10^−1^ to 10^−5^) and 2.5 µL spotted onto Sabouraud plates containing (**A**) sorbitol and sodium chloride for osmotic stress and (**B**) hydrogen peroxide for oxidative stress or. Representative images of three independent experiments with similar results are shown. (**C**) Additional evaluations of *C. auris* resistance to H_2_O_2_ upon contact with *A. castellanii* were performed by the CFU assays. Experiments were repeated three times, with two experimental replicates each. Statistical analyses display the comparison of the average for each group with the untreated controls (*P < 0.05, **P < 0.01, and ***P < 0.001).

Since *C. auris* persistence in the environment is one of its striking characteristics, the resistance of both MMC1 and MMC2 *C. auris* isolates to osmotic stress upon contact with *A. castellanii* or its products was evaluated under two conditions: a hyperosmolar stress with 1 M sorbitol and a hypertonic stress with 1.5 M NaCl. The MMC1 isolate of *C. auris* displayed a slightly more sensitive phenotype to either sorbitol (Fig. 3A, upper left panel) or NaCl (Fig. 3A, lower left panel) following direct contact with *A. castellanii.* In addition, contact with the secreted fractions *A. castellanii* resulted in a mixed response for this isolate, with more resistance to sorbitol and less resistance to NaCl osmotic stressors. Strikingly, the MMC2 isolate exhibited a more resistant phenotype to both osmotic stressors upon direct contact with *A. castellanii* or with its secreted fraction (Fig. 3A, sorbitol, upper right panel, and NaCl, lower right panel).

Regarding the resistance to oxidative stress, the MMC1 isolate of *C. auris* demonstrated relative resistance to H_2_O_2_ 2.5 mM, as evidenced by the growth of untreated PYG controls down to a 10^−4^ dilution. However, direct contact with *A. castellanii* or its secreted fraction showed no impact on the susceptibility of this *C. auris* isolate to H_2_O_2_ by the spot assay (Fig. 3A, left panel). Conversely, for the MMC2 isolate, direct contact with *A. castellanii* trophozoites or its secreted products resulted in a more resistant phenotype of *C. auris* to H_2_O_2_, with yeast growth observed down to 10^−3^ dilutions for both treatments in comparison to 10^−2^ growth observed in the PYG control (Fig. 3B, right panel). As the resistance to oxidative stress is an important feature of *C. auris*, additional evaluations were performed by determining the CFU number upon yeast recovery and exposure to H_2_O_2_ (Fig. 3C). In accordance, both MMC1 and MMC2 isolates, upon direct contact with *A. castellanii*, displayed enhanced resistance to H_2_O_2_ (Fig. 3C, left and right panels, respectively). Furthermore, the *C. auris* MMC2 isolate also displayed enhanced resistance upon contact with secreted products of *A. castellanii* (Fig. 3C, right panel).

### *C. auris* response to pH and thermic stress upon direct or indirect interactions with *A. castellanii*

**Fig 4:**
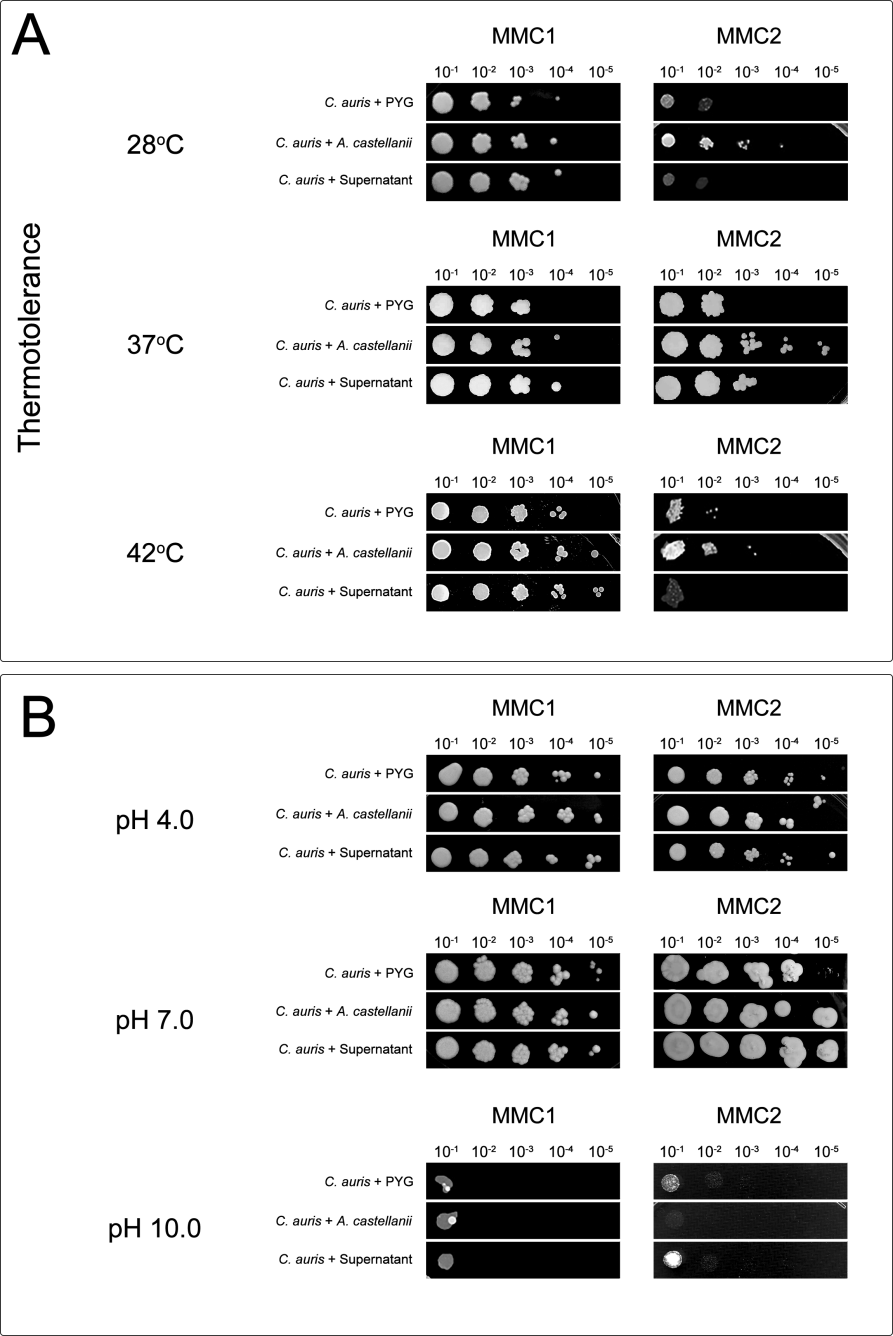
Spot assay for evaluating thermotolerance and pH stress sensitivity of *C. auris* after contact with *A. castellanii* trophozoites or its fractions. After the distinct treatments, recovered yeast cells from each condition were normalized to 0.1 absorbance at 600 nm, further serially diluted 10-folds (10^−1^ to 10^−5^), and 2.5 µL spotted onto Sabouraud plates and incubated at 28°C, 37°C, and 42° for (**A**) thermotolerance or distinct (**B**) pHs. Representative images of three independent experiments with similar results are shown.

Growth of *C. auris* when exposed to additional stressors, such as temperature and pH, was evaluated upon contact with *A. castellanii*. The isolate MMC1 displayed no difference in growth at 28°C upon direct contact with *A. castellanii* (Fig. 4A, left upper panel). However, at 37°C and 42°C, *C. auris* exposed to *A. castellanii* displayed a more thermoresistant phenotype (Fig. 4A, middle and lower left panels, respectively). For the MMC2 isolate, direct contact with *A. castellanii* had a more resistant phenotype at all growth temperatures evaluated (Fig. 4A, right panels).

Regarding the sensitivity to pH, the MMC1 isolate was not impacted by treatment with *A. castellanii* or its fractions (pH 4.0, pH 7.0, and pH 10.0; Fig. 4B, left panels). For the MMC2 isolate, contact with either *A. castellanii* trophozoites or its secreted fractions resulted in a slightly higher resistance at pH 7.0 (Fig. 4B, middle right panel), and a more sensitive phenotype to pH 10.0 was observed upon specific mechanical contact with *A. castellanii* (Fig. 4B, lower right panel).

### *C. auris* expression of virulence-related lipid metabolism factors upon contact with *A. castellanii* or its isolated fractions

**Fig 5:**
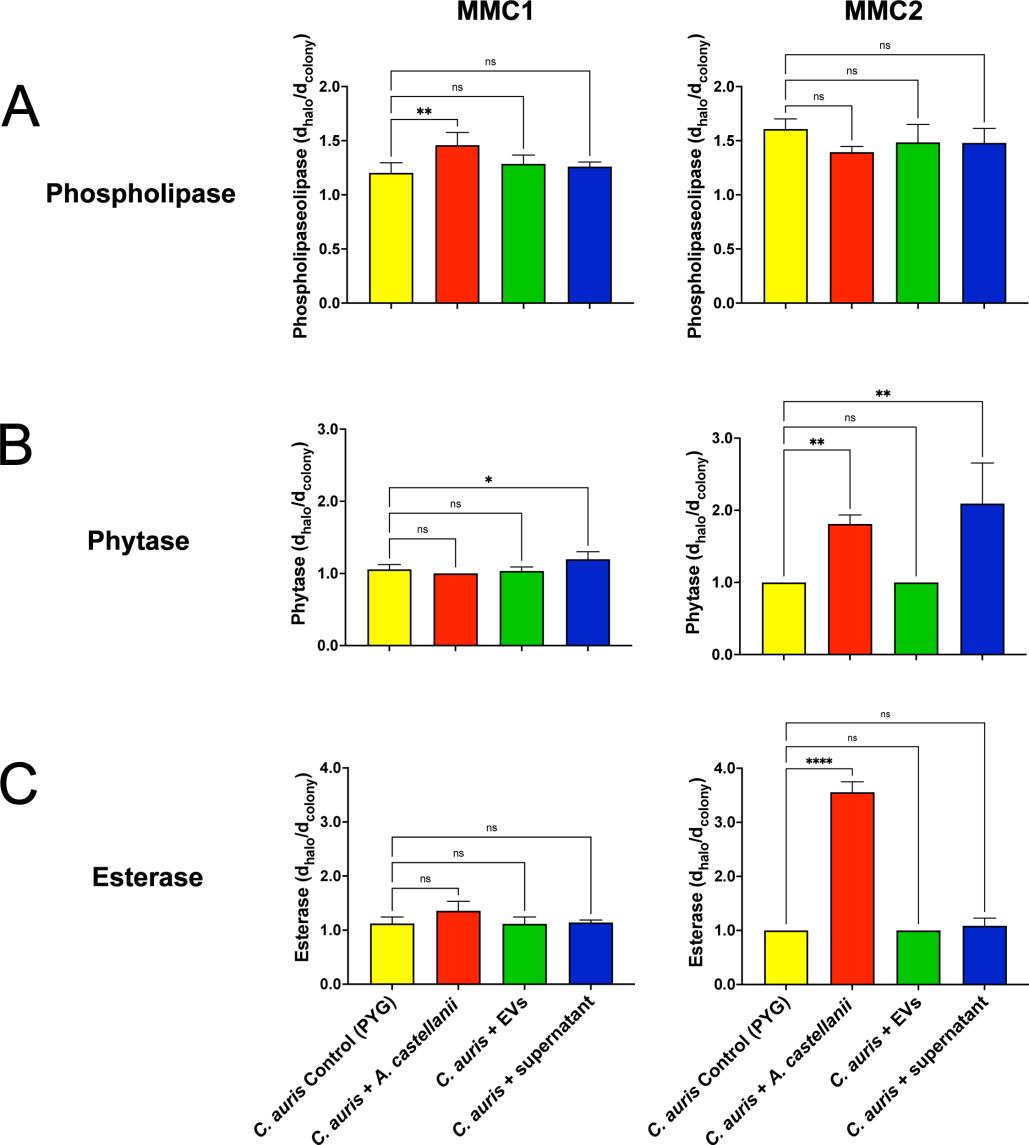
Enzymatic activity of *C. auris* MMC1 (left panels) and MMC2 isolates (right panels after direct contact with trophozoites or indirect contact with EVs or supernatant from *A. castellanii.* Phospholipase and phytase activities were characterized by a precipitation (opaque) and a degradation (translucent) halo around the colony, respectively, whereas esterase activity was given by the formation of a precipitation (opaque) halo. Enzyme activities were compared among conditions for each isolate as the ratio of the diameter of the formed halo and the diameter of the colony. Experiments were repeated three times, with three experimental replicates each. Statistical analyses display the comparison of the average for each group with the untreated controls (*P < 0.05, **P < 0.01, and ****P < 0.0001).

The expression of secreted virulence factors related to lipid metabolism in *C. auris* was evaluated upon direct contact with trophozoites, isolated EVs produced by these protozoa, or the conditioned supernatant containing soluble factors released by *A. castellanii*. Overall, both MMC1 and MMC2 isolates were impacted to some extent by contact with *A. castellanii*. Regarding the expression of phospholipases, the MMC1 isolate demonstrated an increase in the expression of these proteins only upon direct contact with trophozoites (Fig. 5A, left panel). In contrast, the MMC2 isolate showed no significant change in phospholipase expression (Fig. 5A, right panel).

Both isolates of *C. auris* exhibited enhanced expression of phytase upon exposure to soluble fractions secreted by *A. castellanii* (Fig. 5B); only the MMC2 isolate displayed increased phytase expression upon direct contact with trophozoites (Fig. 5B, right panel). Regarding esterase expression, direct mechanical contact of trophozoites with the MMC2 isolate (Fig. 5C, right panel) dramatically increased esterase levels, whereas the MMC1 isolate showed a slight increase (Fig. 5C, left panel).

### *C. auris* expression of virulence-related nutrient acquisition factors upon contact with *A. castellanii* or its isolated fractions

**Fig 6:**
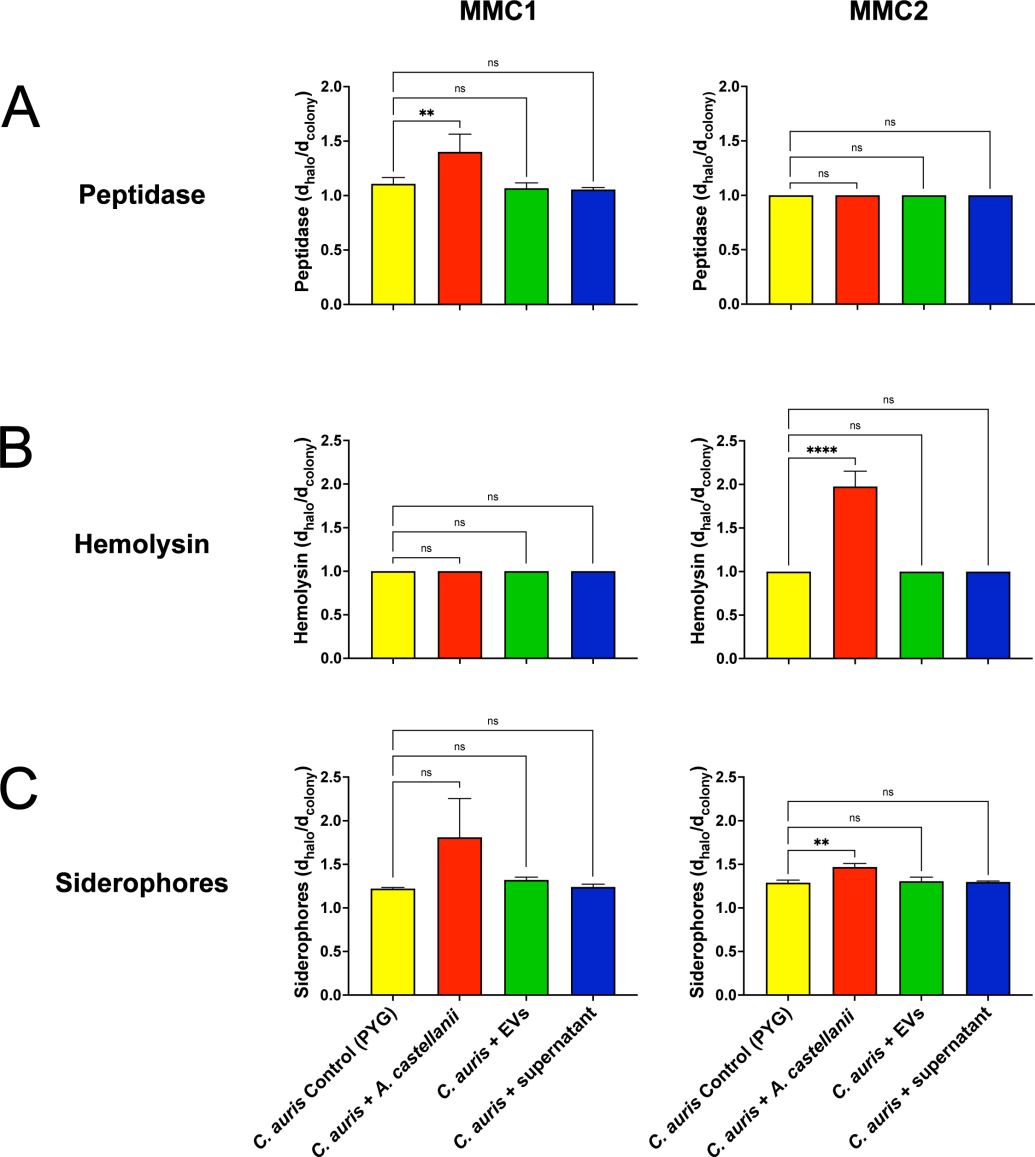
The enzymatic and chelating activity of *C. auris* MMC1 (left panels) and MMC2 isolates (right panels following direct contact with trophozoites or indirect contact with EVs or supernatant of *A. castellanii.* Peptidase and hemolysin activities were characterized by a translucent halo around the colony, whereas siderophore activity was demonstrated by a light green halo. Activities were compared among conditions for each isolate as the ratio of the diameter of the formed halo and the diameter of the colony. Experiments were repeated three times, with three experimental replicates each. Statistical analyses display the comparison of the average for each group with the untreated controls (**P < 0.01 and ****P < 0.0001).

The expression of secreted virulence factors related to nutrient acquisition in *C. auris* was evaluated upon different forms of contact with *A. castellanii*. Regarding peptidase expression, only direct contact with trophozoites increased the expression of this enzyme in the MMC1 isolate (Fig. 6A, left panel); no alterations in peptidase expression were observed in the MMC2 isolate (Fig. 6A, right panel). However, for the MMC2 isolate, hemolysin and siderophore levels increased upon direct contact with trophozoites (Fig. 6B and C, right panels, respectively).

### The direct contact with *A. castellanii* trophozoites enhanced *C. auris* biofilm formation

**Fig 7:**
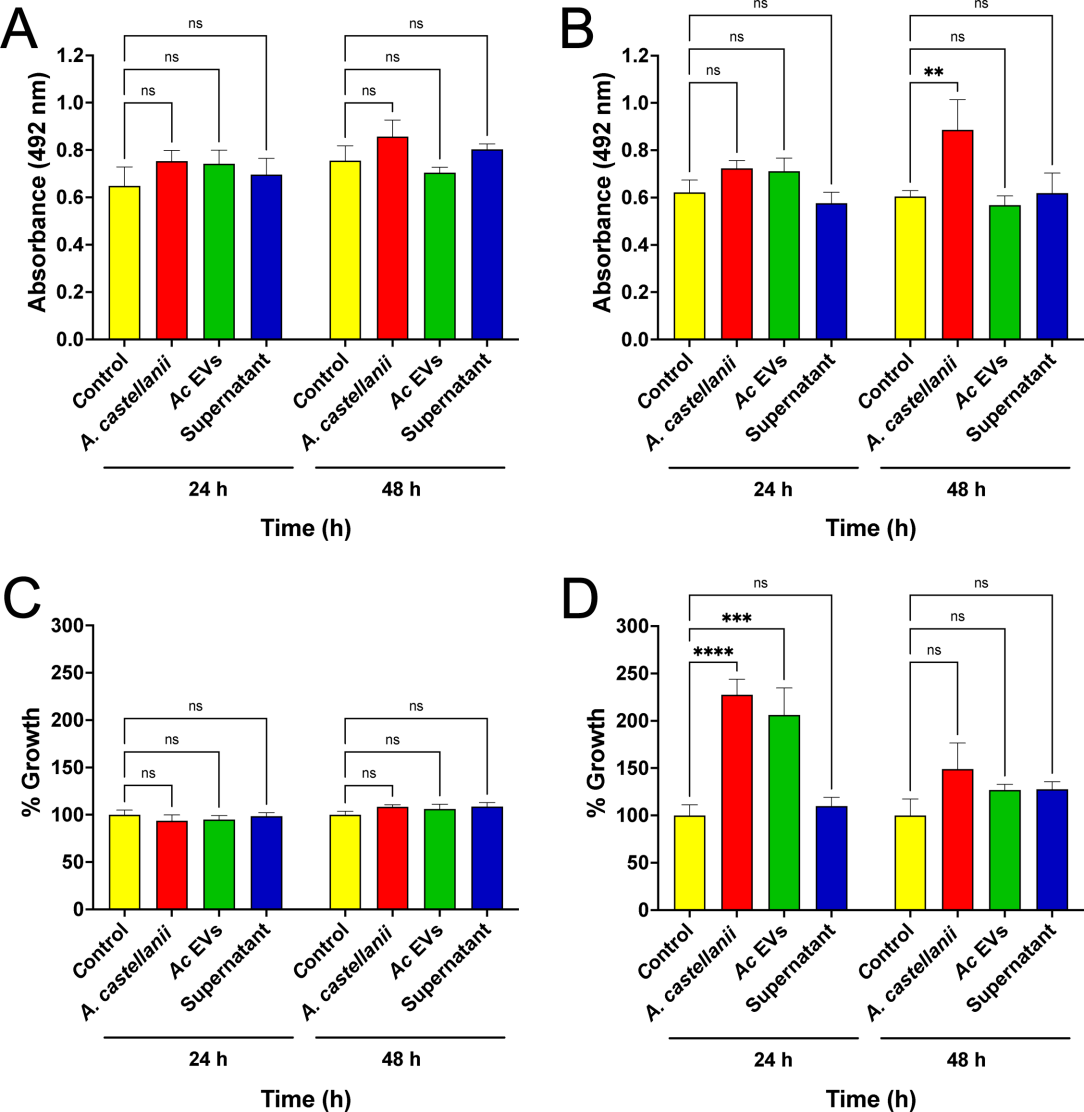
Interactions with *A. castellanii* enhance the formation of biofilms by *C. auris*. After interactions with trophozoites, EVs, or supernatant (soluble proteins) from *A. castellanii*, recovered yeasts were counted and grown under biofilm-forming conditions. (**A and B**) Crystal violet assay to determine the biofilm biomass for the (**A**) MMC1 and the (**B**) MMC2 isolates of *C. auris*. (**C and D**) Metabolic activity by the Alamar blue assay for the (**C**) MMC1 and (**D**) MMC2 isolates of *C. auris*. Experiments were repeated three times. Statistical analyses display the comparison of the average for each group with the untreated controls (**P < 0.01, ***P < 0.001, and ****P < 0.0001).

As EVs from *Candida* spp. or other sources have been shown to impact the *Candida* spp. biofilms formation, we investigated whether direct contact with trophozoites or indirect contact with *A. castellanii* secreted products could impact *C. auris* biofilms formation. While contact with trophozoites did not affect biofilm formation or metabolic activity in isolate MMC1 (Fig. 7A and C, respectively), the isolate MMC2 exhibited enhanced biofilm formation, as evidenced by increased biofilm biomass (Fig. 7B) and its metabolic activity (Fig. 7D). Additionally, indirect contact with *A. castellanii* EVs also promoted biofilm formation by *C. auris* (Fig. 7D).

### Contact of *C. auris* with *A. castellanii* trophozoites enhances susceptibility to amphotericin B due to augmentation of ergosterol levels

**Fig 8:**
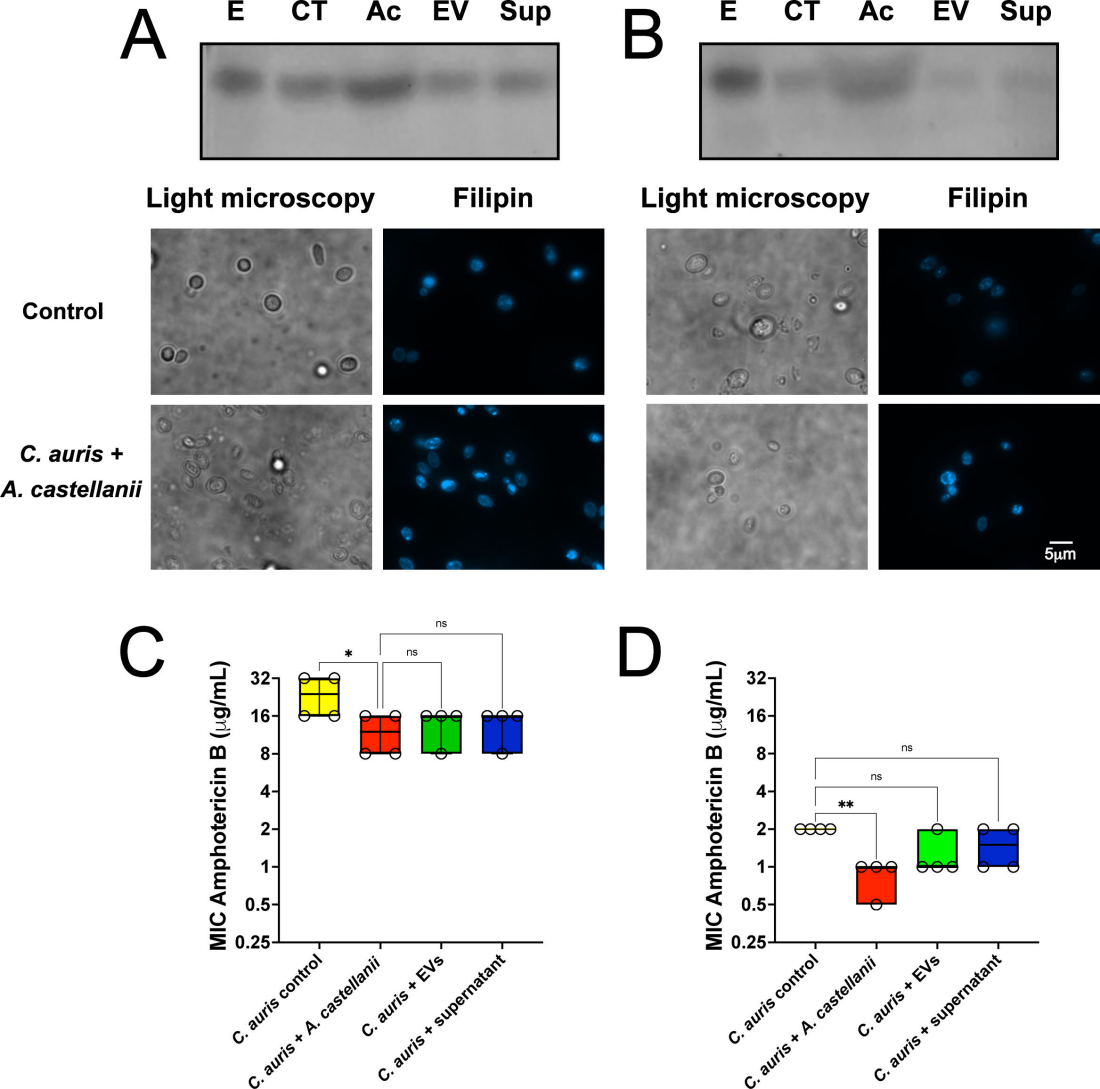
Interactions with *A. castellanii* trophozoites augment ergosterol levels in the (**A**) MMC1 and (**B**) MMC2 isolates of *C. auris*. Lane E, ergosterol standard; CT, *C. auris* grown in PYG medium (control); Ac, *C. auris* grown with trophozoites; EVs, *C. auris* grown with EVs of *A. castellanii;* Sup, *C. auris* grown with soluble fractions of *A. castellanii*. Fluorescent microscopy demonstrates the accumulation of sterol within the cytoplasm, with an intense accumulation of droplets, and on the surface of *C. auris.* (**C and D**) Amphotericin B susceptibility assay for the (**C**) MMC1 and (**D**) MMC2 isolates of *C. auris* upon the distinct treatments with *A. castellanii*. Accumulation of ergosterol led to an increased susceptibility to amphotericin B for both isolates of *C. auris*. Experiments were repeated in biological quadruplicates. Statistical analyses display the comparison of each group with the untreated controls (*P < 0.05 and **P < 0.01).

Ergosterol levels in recovered *C. auris* were evaluated upon various treatment conditions of direct or indirect contact with *A. castellanii*. Both MMC1 and MMC2 isolates exhibited increased ergosterol levels upon direct contact with *A. castellanii* (Fig. 8A and B, respectively), while indirect contact with *A. castellanii* EVs or its conditioned supernatant displayed no effect on ergosterol levels. Strikingly, fluorescence microscopy using the filipin probe specifically targeting fungal sterol revealed an accumulation of ergosterol within the yeast cytoplasm and plasma membrane upon *C. auris* direct contact with *A. castellanii*. Due to the increase in these sterol levels, we proceeded to evaluate the susceptibility of *C. auris* to amphotericin B, which targets fungal sterol. For the MMC1 isolate, direct contact with *A. castellanii* increased the susceptibility to amphotericin B (*P* = 0.014, Fig. 8C). Moreover, an enhanced susceptibility phenotype was also observed for the MMC2 *C. auris* isolate following contact with *A. castellanii* (*P* = 0.0062; Fig. 8D).

For both isolates, recovered yeasts previously subjected to direct contact with *A. castellanii* were cultured for six passages (6 times 48 h culture) in plain PYG medium to assess the time span of ergosterol enhancement. For the *C. auris* MMC1 isolate, statistically increased ergosterol levels were detected up to the first passage (Fig. S2A). In contrast, ergosterol levels of the MMC2 isolate remained elevated after the second passage (Fig. S2B).

### Contact of *C. auris* with *A. castellanii* trophozoites induces a more tolerant *C. auris* phenotype to itraconazole

**Fig 9:**
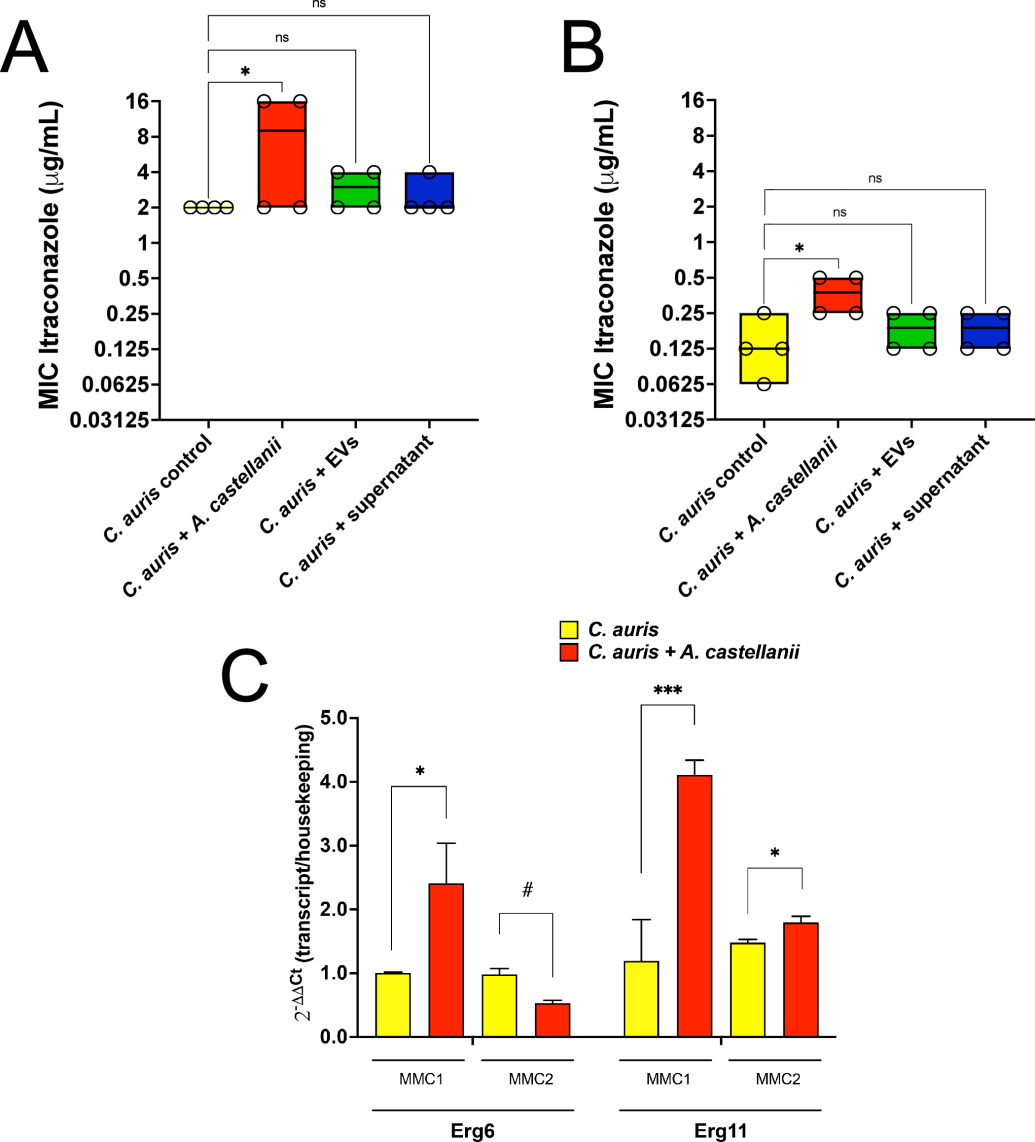
*C. auris* contact with *A. castellanii* results in enhanced resistance to itraconazole. (**A and B**) Itraconazole susceptibility testing for the (**A**) MMC1 and (**B**) MMC2 isolates of *C. auris*. (**C**) Real-time PCR for the expression of the Erg 6 and Erg 11 genes by *C. auris* upon direct interactions with *A. castellanii*. Both transcript levels were determined upon normalization with the tubulin housekeeping gene for each condition. Experiments were repeated in biological quadruplicates. Statistical analyses display the comparison of each group with the untreated controls (*P < 0.05 and **P < 0.01; #*P* < 0.05, decreased expression, recovered *C. auris* vs control).

Distinct treatments of *A. castellanii* resulted in varying susceptibility profiles of recovered *C. auris* yeast to itraconazole. Direct contact of MMC1 isolate with *A. castellanii* increased the MIC to itraconazole compared to untreated *C. auris* (*P* = 0.029; Fig. 9A). Similarly, the MMC2 isolate demonstrated a more tolerant phenotype to itraconazole, growing in concentrations up to four times higher than untreated *C. auris* (*P* = 0.032; Fig. 9B). Both isolates exhibited similar behavior regarding increased expression of the lanosterol 14-α-demethylase enzyme (Erg 11), which correlates with increased resistance to azoles (Fig. 9C). Regarding the Erg 6 expression level, which is involved in consuming toxic ergosterol intermediates, only the MMC1 isolate displayed increased expression levels, whereas the Erg 6 transcripts were downregulated in the MMC2 isolate.

### Contact of *C. auris* with *A. castellanii* trophozoites affected tolerance to caspofungin

**Fig 10:**
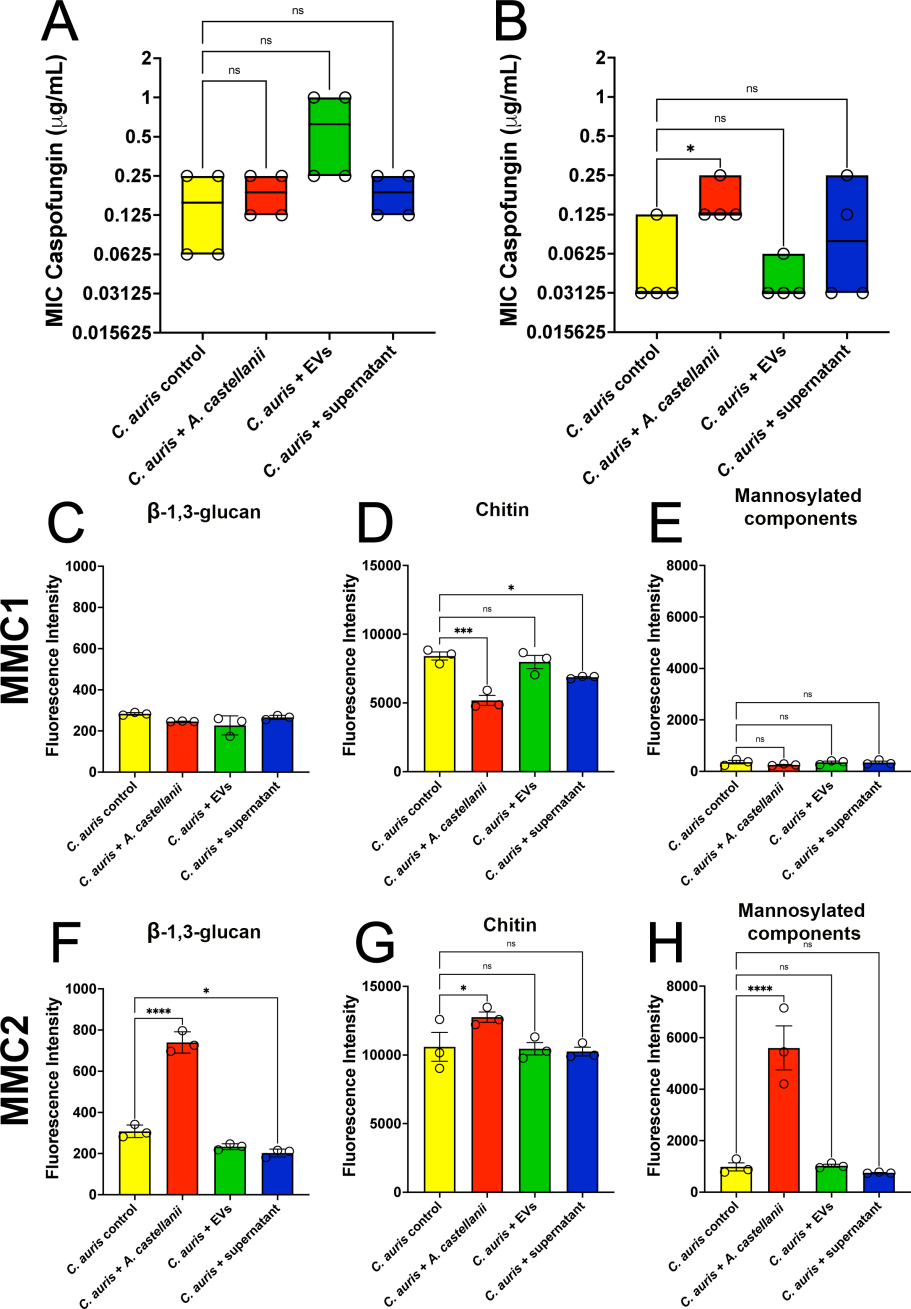
*C. auris* contact with *A. castellanii* results in slightly enhanced tolerance to caspofungin. (**A and B**) Caspofungin susceptibility testing for the (**A**) MMC1 and (**B**) MMC2 isolates of *C. auris*. Experiments were repeated in biological quadruplicates (**C–E**) Assessment of the levels of (**C**) β-1,3-glucan, (**D**) chitin, and (**E**) mannosylated components for the MMC1 isolate of *C. auris*. (**F–H**) Assessment of the levels of (**F**) β-1,3-glucan, (**G**) chitin, and (**H**) mannosylated components for the MMC2 isolate of *C. auris*. Flow cytometry was performed in triplicate. Statistical analyses display the comparison of each group with the untreated controls (*P < 0.05, ***P < 0.001, and ****P < 0.0001).

Recovered *C. auris* yeasts from distinct contact forms with *A. castellanii* were tested for susceptibility to caspofungin. Both MMC1 and MMC2 *C. auris* isolates recovered after contact with trophozoites displayed increased tolerance to caspofungin (Fig. 10A and B, respectively) that was more pronounced for the MMC2 isolate, which had a ~4-fold increase in MIC, although no statistical significance was observed. Contact with trophozoites did not significantly alter MMC1 isolate levels of β-1,3-glucan (Fig. 10C), chitin (Fig. 10D), or mannosylated components (Fig. 10E). However, the interaction of the MMC2 isolate with trophozoites significantly increased levels of β-1,3-glucan (Fig. 10F), chitin (Fig. 10G), and mannosylated components (Fig. 10H), corroborating the higher tolerance to caspofungin observed. The levels of the transcripts for the Fks1 and Fks2 subunits of the β-1,3-glucan synthase and the Hog 1 protein were evaluated by RT-qPCR for both MMC1 and MMC2 isolates of *C. auris* upon recovery from co-cultivation with trophozoites of *A. castellanii* (Fig. S3A and B, respectively). The Fks1p was downregulated in the MMC2 isolate upon passage through *A. castellanii*, whereas the Hog1 protein was downregulated in both isolates upon passage through amoeba.

### Contact with *A. castellanii* trophozoites impacts the virulence of *C. auris* in a Lepidoptera *Galleria mellonella* model

**Fig 11:**
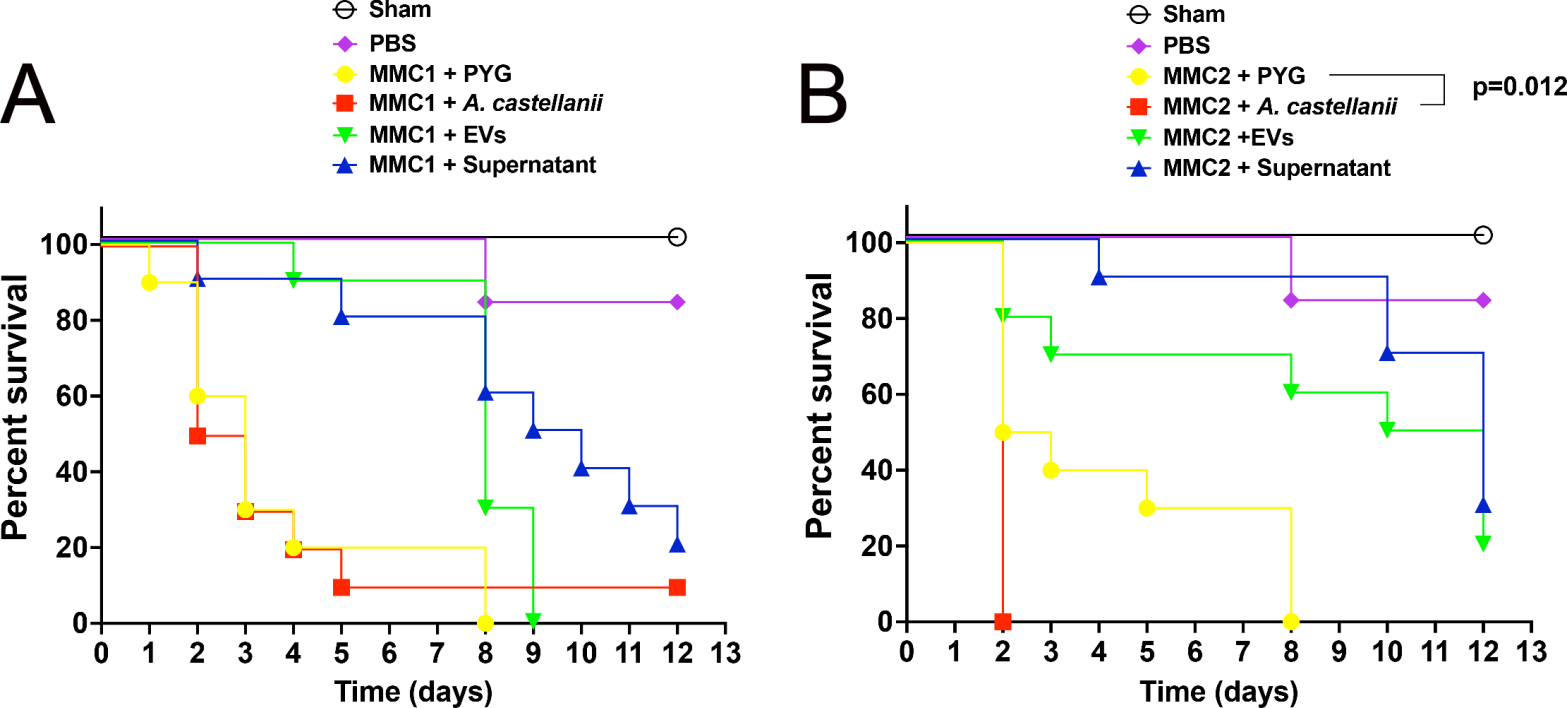
Interactions of *C. auris* with *A. castellanii* impacted the yeast virulence in *G. mellonella* infection models. (**A and B**), Survival curves for *G. mellonella* upon infection with recovered yeasts from the distinct treatments of *C. auris* with *A. castellanii* were compared for the isolates (**A**) MMC1 and (**B**) MMC2. No statistical significance was observed for the comparisons within the MMC1 isolate, but for the MMC2 isolate, direct contact with *A. castellanii* resulted in a more virulent phenotype of *C. auris* (*P < 0.05).

The effects of *C. auris* interactions with *A. castellanii* or its fractions were evaluated in a *G. mellonella* survival model. The MMC1 isolate fully eradicated the *G. mellonella* larvae within 8 days (Fig. 11A). Direct contact with *A. castellanii* had no discernible impact on virulence compared to untreated *C. auris* controls. For the MMC2 isolate, although untreated *C. auris* also killed all larvae by day 8, direct contact with *A. castellanii* resulted in a more virulent *C. auris* phenotype, as all larvae died within 2 days after challenge with the exposed *C. auris* yeasts (Fig. 11B, *P* = 0.012 compared to controls).

## DISCUSSION

The vast majority of human pathogenic fungi are free-living organisms causing “accidental” exogenous infections upon acquisition from environmental sources (59). These organisms likely acquire pathogenic traits in environmental niches that mirror certain aspects of the human body, undergoing metabolic and physicochemical pressures that could influence fungal virulence (17, 59, 60). Consequently, it is essential to investigate how pathogenic fungi adapt to diverse stress scenarios they may encounter in the environment, and potentially in clinical settings. In this regard, the emerging multidrug-resistant fungal pathogen *C. auris* is believed to have developed the ability to overcome challenging environmental stressors that have enabled its environmental persistence to become an often-associated hospital-acquired infection and the adaptation to host-imposed infection responses that result in the species’ ability to effectively infect and colonize humans. In this context, environmental interactions with unicellular hosts such as *A. castellanii* or other FLAs are thought to have shaped the evolution and adaptation of pathogens that escape degradation by these environmental phagocytic predators. The selective pressure exerted by such microbial-environmental predator interactions, as highly conserved processes to eukaryotic phagocytes, may explain why these amoeba-resistant organisms have expanded their range of potential hosts, including mammals (33). In this study, we investigate the interaction dynamics between distinct isolates of *C. auris,* the MMC1 fluconazole-resistant, and the MMC2 fluconazole-sensitive isolate (34) with *A. castellanii*, aiming to elucidate the potential mechanisms and their implications for fungal adaptation to amoeba predation, shedding light on the impact of these interactions through the assessment of various aspects of *C. auris* physiology, which could directly impact the fungal susceptibility to clinically used antifungals, and virulence. Both isolates were chosen based on previous phenotypic characterization. The MMC1 isolate could normally grow at fluconazole concentrations up to 1,000 µg/mL, which complicated further evaluations on the impact of interactions with *A. castellanii* on the susceptibility of this azole, particularly due to the higher levels of expression of the efflux pump CDR1 and the Erg 6 enzyme (34). Besides higher resistance to fluconazole, the MMC1 *C. auris* isolate was also more tolerant to amphotericin B and caspofungin (34). The MMC1 isolate also produced larger EVs, with higher levels of ceramide Cer (d18:1/2:0) and unsaturated phosphatidylglycerols (PGs 18:1/0:0 and 18:2/0:0) in comparison to the MMC2 isolate. The MMC1 EVs could also promote more adhesion of yeasts to epithelial cells and more activation of dendritic cells than the ones from the MMC2 isolate (61). Additionally, the MMC1 isolate was also able to melanize in the presence of catecholamines, as opposed to the MMC2 *C. auris* isolate (62, 63).

Initially, we examined the kinetics of interaction between *C. auris* and *A. castellanii,* focusing on the variables time and MOI. The calculated mathematical parameters indicated that, for both isolates, the variable time had no discernable impact on the fungal-amoeba interaction. However, regarding the variable MOI, interactions were similar for both isolates up to the ratio 5 fungi:1 amoeba; at 10:1, the MMC2 *C. auris* association rate to *A. castellanii* continued increasing, regardless of the time evaluated. In contrast, the MMC1 *C. auris* isolate demonstrated an accentuated drop in the interaction rates at the 10:1 MOI, primarily due to the rounding of *A. castellanii* trophozoites and subsequent killing of the amoeba by the fungus.

A previous report by Hubert et al*.* (64) described the capacity of *C. auris* to survive in contact with the FLAs *A. castellanii* and *Vermamoeba vermiformis* in tap water, with an initial decline in fungal CFUs within the first 48 h of co-incubations, followed by an increased *C. auris* CFU numbers over time. Additionally, exposure to supernatants from either FLA promoted fungal growth more prominently than growth in direct contact with trophozoites of either amoeba. However, the viability of *C. auris* upon direct incubations with *A. castellanii* was not assessed. The examination of viability and growth capacity of both fluconazole-resistant (MMC1) and -sensitive (MMC2) isolates of *C. auris* was also a crucial step in our evaluations, as the adaptation and selection imposed by *A. castellanii* contact depends on fungal survival and adaptation to these phagocytes. In our studies, the two *C. auris* isolates demonstrated the ability to survive and replicate within the intracellular milieu of *A. castellanii*, albeit with distinct viability patterns over time up to 48 h, with the MMC1 exhibiting greater resistance to amoeba killing. As both isolates remained viable during co-culture with amoeba, exposure to the selective pressure in the trophozoite intracellular milieu could lead to a multifactorial stress response, possibly altering metabolism and the expression of *C. auris* virulence attributes, which was further evaluated.

In this regard, both *C. auris* isolates, MMC1 and MMC2, revealed differential growth curve kinetics upon exposure to direct or indirect interactions with *A. castellanii* or its secreted products. While both isolates grew similarly in RPMI medium (with ~5-h doubling times), yeasts recovered from contact with *A. castellanii* trophozoites or their secreted products demonstrated accelerated growth, with a reduction of approximately 40% in the yeast doubling time for the MMC1 and MMC2 *C. auris* isolates. Notably, existing literature has not made such comparisons regarding fungal growth following *A. castellanii* contact and recovery/isolation, focusing instead on their growth capacity within amoeba (24, 25, 37, 64). In fact, enhanced growth capacity of *C. neoformans* upon passages in the social amoeba *Dictyostelium discoideum* has been reported (65).

Intrinsically, *C. auris* isolates may exhibit tolerance to various stressors, including osmotic, oxidative, and thermal stresses, which may contribute to their environmental survival, spread, and dynamic transmission (42, 66). Similar to other *Candida* species, *C. auris* appears to tolerate a wide range of pH, but displays greater resistance to alkaline environments, including diluted hypochlorite, quaternary ammonia sanitizing solutions, and chlorhexidine solutions (67, 68), enhancing its persistence on patient skin, environmental surfaces, and hospital linen for several days (67, 69–72).

Considering that the development of *C. auris* resistance to harsh conditions may depend on physical and physiological context, we explored the response of *C. auris* to various stressors, including oxidative, osmotic, thermal, and pH, upon interaction with *A. castellanii*. Overall, MMC1 was minimally impacted by the interaction with *A. castellanii*, displaying a higher sensitivity to osmotic stress (sorbitol and NaCl) and limited resistance to thermal stress at 37°C and 42°C. In contrast, direct contact with *A. castellanii* trophozoites increased significantly the resistance of the *C. auris* MMC2 isolate to oxidative, osmotic, and thermal stress. These data highlight the distinct nature of interactions between the two *C. auris* isolates and *A. castellanii*, suggesting a complex interplay between fungal and amoebal factors in modulating stress responses.

Stress responses in *Candida* species are an area of great scientific interest (73). Recent studies have implicated the protein kinase Hog1 in mediating stress resistance in *Candida*, including *C. auris* (74, 75). The protein Hog1 interacts with the Mkc1 protein as part of the MAPK cascade, playing crucial roles in infection and fungal virulence by regulating cell wall integrity and metabolic pathways, cell cycle, and distinct stress responses (76, 77). Deletion of Hog1 in *C. auris* results in higher tolerance to caspofungin and alterations in cell wall composition, with higher mannan and chitin cell wall levels (74, 75, 78). Hog1 transcripts were downregulated by *C. auris* upon co-cultivation with *A. castellanii*. Specifically for the MMC2 strain, higher content of cell wall polysaccharides, including β-1,3-glucan, chitin, and mannosylated components, were observed, which could consist of a compensatory mechanism leading to higher tolerance to caspofungin and resistance to other stressors. Similarly, *in vitro* induction of tolerance to caspofungin using subinhibitory concentrations of the drug led to an increase in the cell wall chitin levels (79).

*C. auris* shares many virulence and fitness attributes with *C. albicans*, including the secretion of proteases and lipases, the expression of siderophores, and biofilm formation, among others (66, 80, 81). Upon interaction with *A. castellanii*, the expression of enzymes related to lipid metabolism and nutrient acquisition varied between the MMC1 and MMC2 *C. auris* isolates and interaction conditions, suggesting distinct and intricate regulatory mechanisms underlying fungal virulence for each isolate. The MMC1 isolate enhanced the secretion of phospholipases and peptidases upon direct interactions with trophozoites of *A. castellani*i, whereas the MMC2 isolate upregulated the expression of phytase, esterase, hemolysin, and siderophores, demonstrating a more adaptable phenotype for *A. castellanii* selective pressure. Additionally, the MMC2 exhibited enhanced biofilm formation and altered expression of virulence-related factors upon direct contact with trophozoites or exposure to *A. castellanii*-released EVs.

Recently, Bing et al*.* (82) reported the occurrence of a new *C. auris* phenotype *in vivo*, upon the accumulation of *de novo* point mutations in several genes involved in cell division, cytokinesis, cellular polarization, budding, and cell wall integrity, yielding a rapid evolution to a multicellular aggregative morphology in murine systemic infection models. We cannot rule out that such similar pressure may occur within the intracellular environment of *A. castellanii*, which is yet to be determined.

Both the resistant and sensitive isolates of *C. auris* displayed increased ergosterol levels upon interaction with *A. castelanii*, leading to enhanced susceptibility to amphotericin B. Notably, the MMC2 isolate demonstrated a more pronounced effect, with up to twofold lower MICs compared to controls. The ergosterol increase upon a single passage through *A. castellanii* seemed a transitory phenomenon, lasting for at least two additional *C. auris* subcultures in the PYG medium. Increased ergosterol abundance has been associated with higher resistance to several stressors in *Saccharomyces cerevisiae*, including temperature and oxidative stress (83, 84), consistent with our results. However, in *S. cerevisiae*, increased ergosterol levels were correlated with decreased susceptibility to osmotic stress (84), as observed with the MMC1 isolate. In contrast, the MMC2 *C. auris* isolate, this hypersensitivity could have been overcome due to the overall increase of polysaccharide levels, including chitin and β−-,3-glucans on the cell wall of the MMC2 isolate upon interaction with *A. castellanii* trophozoites, also increasing tolerance to caspofungin (79).

Since both isolates demonstrated susceptibility to itraconazole, we elected this azole for further testing. However, interactions between *C. auris* and *A. castellanii* conferred a higher tolerance to this drug. However, evaluating the scenario of the possible mechanisms involved, the two *C. auris* isolates demonstrated increased expression of the Erg 11, which converts lanosterol to 14-α-demethyllanosterol, ensuring continued ergosterol biosynthesis despite drug presence. However, the two isolates differed regarding the expression of Erg 6, which acts downstream in the ergosterol biosynthesis pathway converting zymosterol into fecosterol. Alternatively, Erg 6 is also involved in the first step of the alternative pathway, additionally playing an important role in azole resistance due to the conversion of lanosterol into eburicol and detoxification from the accumulated lanosterol amid Erg 11 inhibition by azoles (50). In essence, an increase in Erg 11 expression could represent a multifaceted strategy employed by fungal cells during interactions with amoeba to counter azole-induced stress, sustain membrane integrity, and maintain vital cellular functions despite azole exposure. A possible mechanism involved in the upregulation of ergosterol biosynthesis enzymes, as observed in *C. albicans*, would involve a gain-of-function UPC2 mutation, resulting in increased activation of this major transcriptional regulator (85). However, to date, activating mutations in the *C. auris UPC2* ortholog (B9J08_000270), alone or in combination with *ERG11* mutations, have not been reported in *C. auris* clinical isolates (86). Furthermore, we cannot rule out that interactions with *A. castellanii* can lead to overexpression of efflux pumps by *C. auris*. Increased resistance to azoles has been observed for *C. albicans* upon interactions with *A. castellanii* trophozoites, with non-synonymous changes in single nucleotide polymorphism (SNP), indels, structural variations, and copy number variations in *C. albicans* genomes; however, none of them related to the aforementioned genes (29).

The impact of the *A. castellanii* trophozoites interactions with *C. auris* on fungal virulence was also assessed in a *G. mellonella* infection model. *C. auris* isolate-specific effects on larval survival were observed, highlighting the potential role of amoebal interactions in modulating fungal virulence, with a greater impact occurring with the MMC2 isolate. This raises the possibility that the MMC2, as a non-aggregative and potentially less virulent phenotype of *C. auris*, could undergo more phenotypical changes imposed by the intracellular environment of the trophozoites. In contrast, the MMC1 isolate could intrinsically be a more virulent phenotype, experiencing less impact by the stressors imposed by the interactions with *A. castellanii*.

In summary, our study provides new comprehensive insights into the ecological and evolutionary dynamics of fungal-amoebal interaction. The elucidation of the complex interplay between *C. auris* and *A. castellanii*, as both might occupy overlapping niches in the environment, shed light on the diverse mechanisms underlying fungal adaptation and susceptibility to antifungal agents and virulence, and could contribute to our understanding of how *C. auris* could have developed strategies to become a multidrug-resistant and a resilient pathogen, with direct implications in the persistent in the nosocomial settings and human host infections (33). By adopting a One Health perspective and implementing comprehensive surveillance, infection control measures, and environmental monitoring, we can better understand and manage the impact of *C. auris* on human and animal health, while also addressing its environmental adaptation and interaction (87).

## SUPPLEMENTAL MATERIAL

Fig. S1 spectrum.01746-24-s0001.tif Growth kinetics of *C. auris* after their recovery from direct interactions with *A. castellanii* or indirect interactions with conditioned supernatant.

Fig. S2 spectrum.01746-24-s0002.tif Sterol levels of *C. auris* yeasts were increased after interacting with *A. castellanii* trophozoites and the levels were initially maintained after passage in PYG medium.

Fig. S3 spectrum.01746-24-s0003.tif Real-time PCR for the expression of the Fks1, Fks2, and Hog1 genes by *C. auris* upon direct interactions with *A. castellanii*.

ASM does not own the copyrights to Supplemental Material that may be linked to, or accessed through, an article. The authors have granted ASM a non-exclusive, world-wide license to publish the Supplemental Material files. Please contact the corresponding author directly for reuse.
