# Supplementary figures and images for: Interactions of the emerging fungus Candida auris with Acanthamoeba castellanii reveal phenotypic changes with direct implications on the response to stress and virulence
Source: Microbiol Spectr. 2024 Dec 17;13(2):e01746-24. doi: 10.1128/spectrum.01746-24 (PMC11792492; doi:10.1128/spectrum.01746-24)

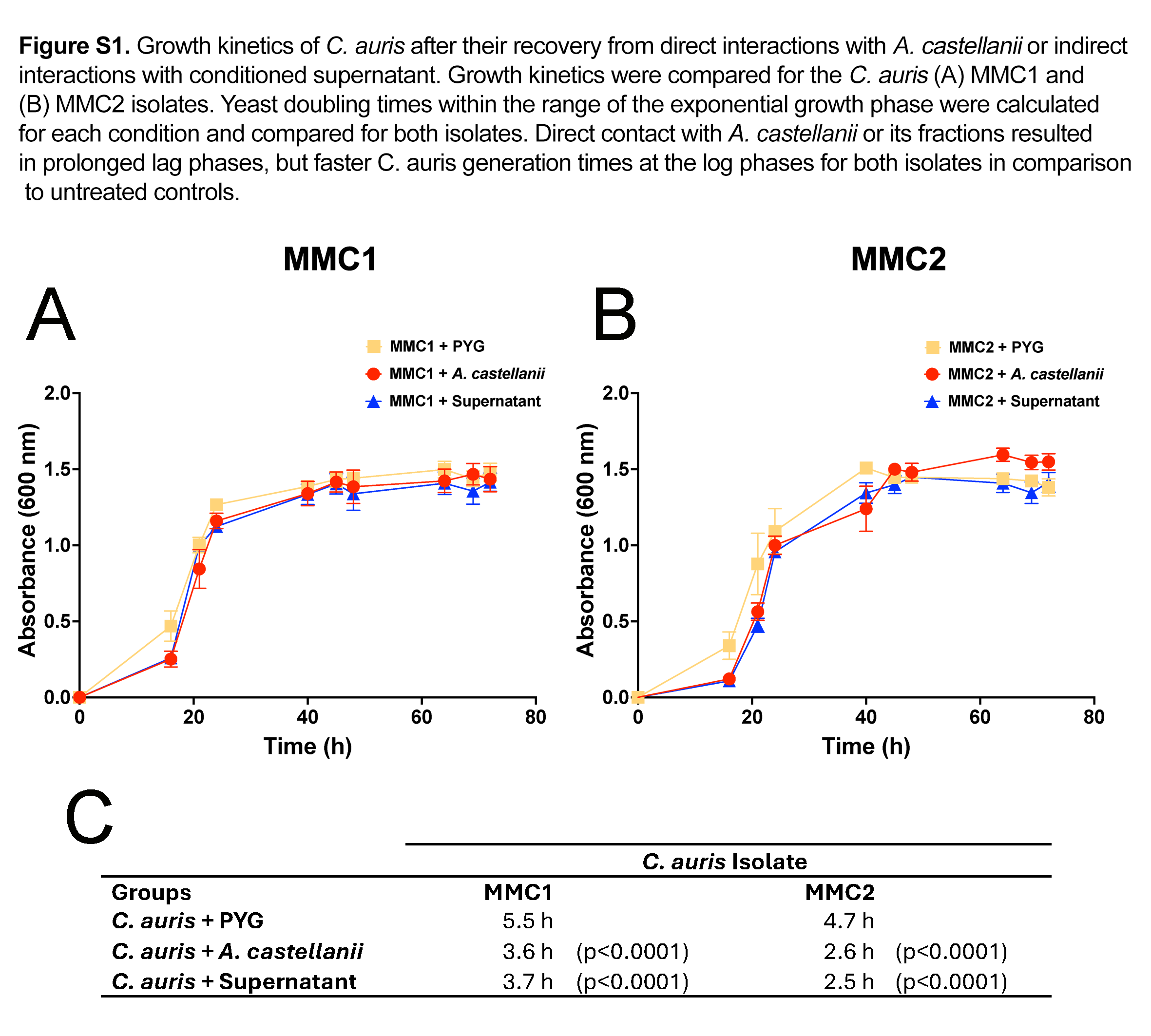

Supplement: Fig. S1 — Growth kinetics of C. auris after their recovery from direct interactions with A. castellanii or indirect interactions with conditioned supernatant. [file spectrum.01746-24-s0001.tif]

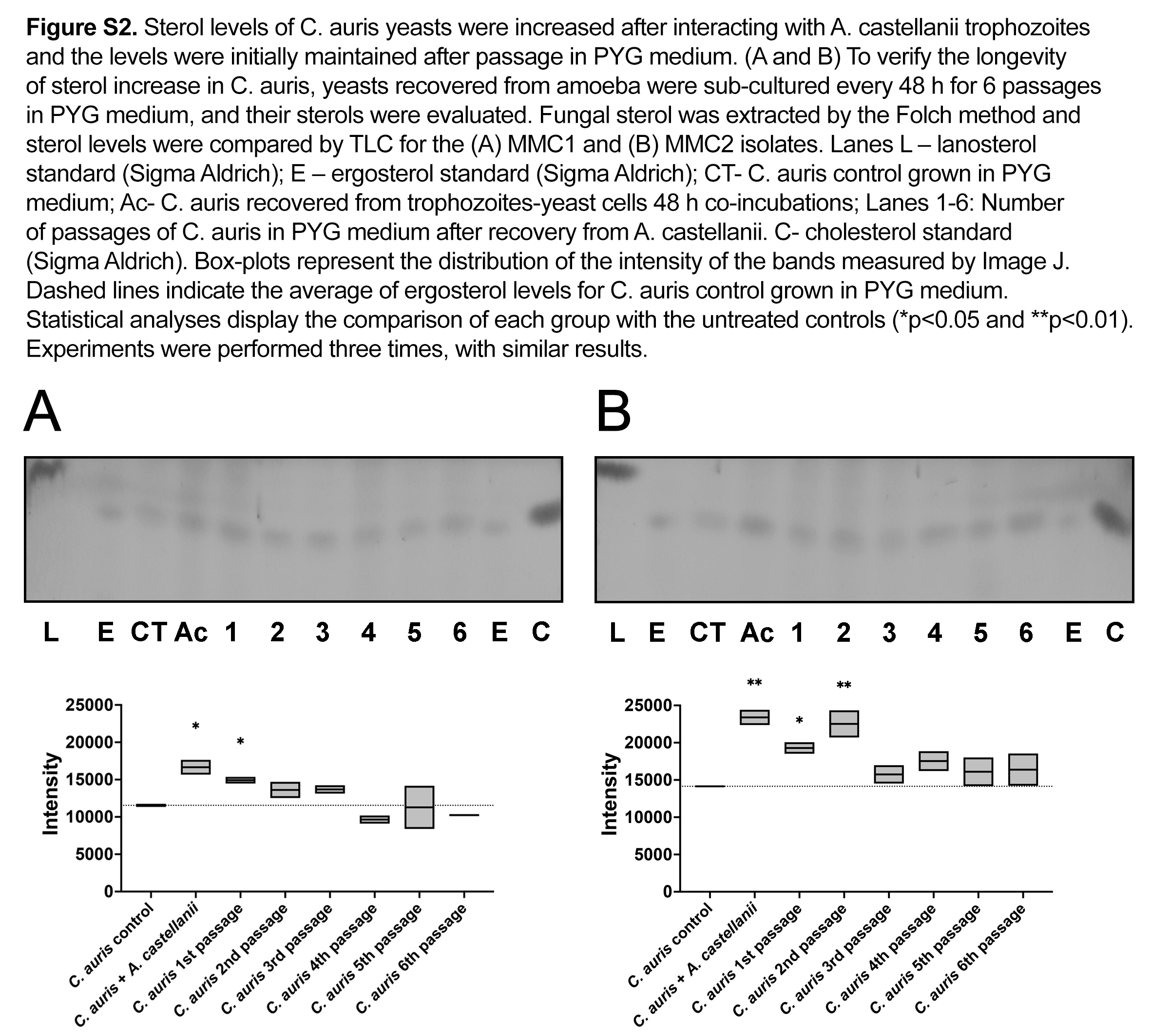

Supplement: Fig. S2 — Sterol levels of C. auris yeasts were increased after interacting with A. castellanii trophozoites and the levels were initially maintained after passage in PYG medium. [file spectrum.01746-24-s0002.tif]

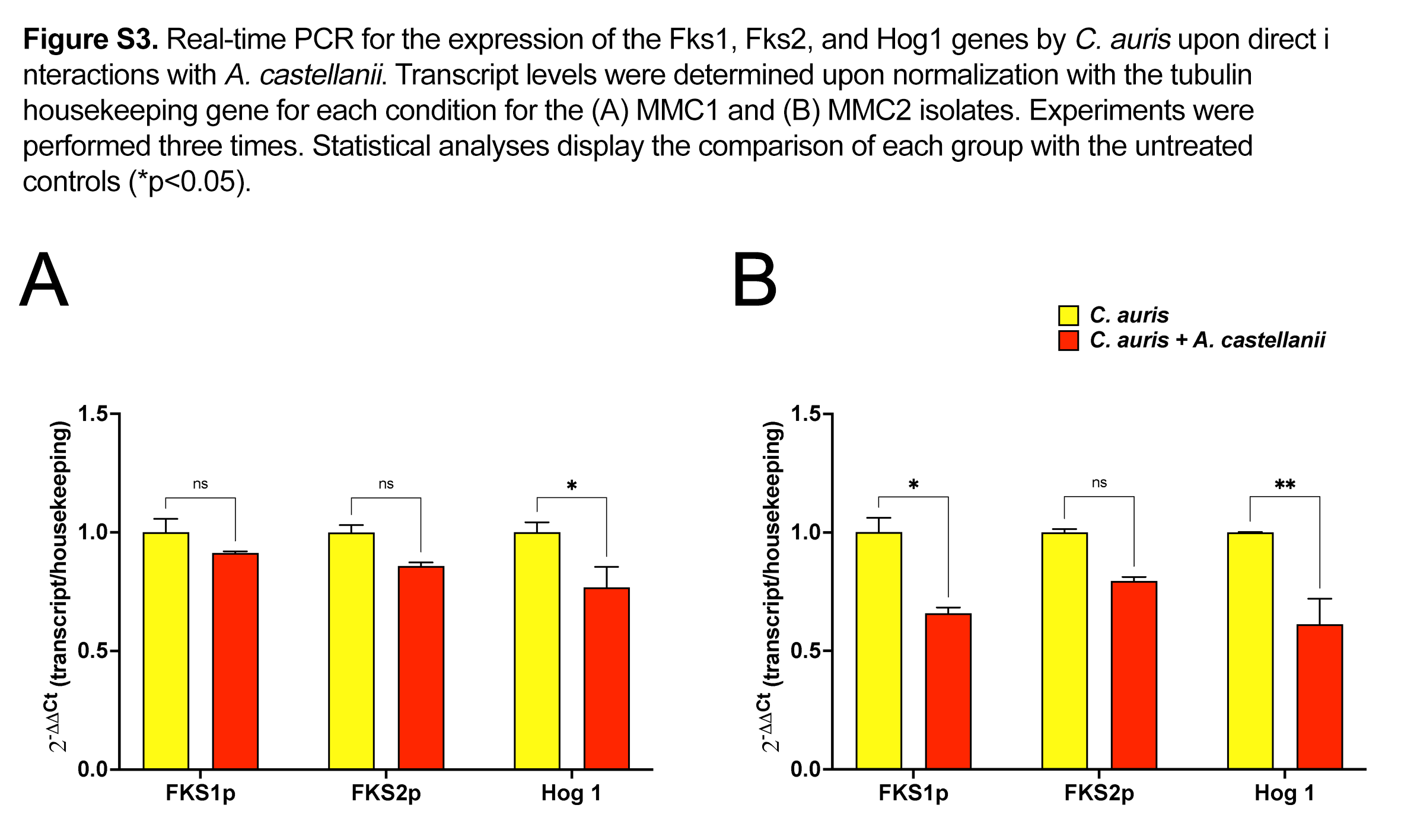

Supplement: Fig. S3 — Real-time PCR for the expression of the Fks1, Fks2, and Hog1 genes by C. auris upon direct interactions with A. castellanii. [file spectrum.01746-24-s0003.tif]
